# Supplementary material for: Interplay between structural hierarchy and exciton diffusion in artificial light harvesting
Source: Nat Commun. 2019 Oct 10;10:4615. doi: 10.1038/s41467-019-12345-9 (PMC6787233; doi:10.1038/s41467-019-12345-9)
Supplement: Supplementary file 1 — Supplementary Information [file 41467_2019_12345_MOESM1_ESM.pdf]

Supplementary Information

Interplay between Structural Hierarchy and Exciton Diffusion in Artificial Light Harvesting

Kriete et al.

Supplementary Note 1: Calculation of Exciton Densities

The exciton densities, i.e., the number of excitons (N_{e}) normalized by the number of molecules (N_{m}) in the focal volume, were computed as outlined elsewhere[1]. The formula that was used for computation is given as:

$$\frac{N_{\mathrm{e}}}{N_{\mathrm{m}}}=\frac{\Delta E}{hc_0}\left(\frac{\int I_{\mathrm{pump}}(x,y)I_{\mathrm{probe}}(x,y)\mathrm{d}x\mathrm{d}y}{\int I_{\mathrm{pump}}(x,y)\mathrm{d}x\mathrm{d}y\int I_{\mathrm{probe}}(x,y)\mathrm{d}x\mathrm{d}y}\right)\left(\frac{\int I_{\mathrm{pump}}(\lambda)\,\lambda\left(1-10^{-\mathrm{OD}(\lambda)}\right)\mathrm{d}\lambda}{\int I_{\mathrm{pump}}(\lambda)\mathrm{d}\lambda}\right)\left(\frac{1}{cN_{\mathrm{A}}d}\right) \tag{1}$$

Here, ΔE is the pulse energy, h the Planck constant, c_0 the speed of light in vacuum and N_{A} the Avogadro constant. The first bracketed factor describes the spatial overlap of the pump transverse beam profile $I_{\mathrm{pump}}(x,y)$ and the probe beam profile $I_{\mathrm{probe}}(x,y)$ at the sample position, while the second bracketed factor accounts for the spectral overlap of the excitation spectrum $I_{\mathrm{pump}}(\lambda)$ with the sample absorption spectrum at a given optical density $\mathrm{OD}(\lambda)$. Finally, the last bracketed factor counts the number of molecules in the focal volume in the denominator. The latter is proportional to the molar concentration of the sample c and the thickness of the microfluidic channel d. The uncertainty of the exciton density was computed via propagation of uncertainty of all relevant input parameters.

In the case of complete nanotubes, the exciton density is considered identical for the inner and outer layer. At a sufficiently low optical density of the sample and assuming similar excitation fluences for both tubes (Figure 1b in the main text), the number of excitons scales with the absorption of the respective tube. The latter in turn scales with the number of molecules in each

layer (Supplementary Note 6.1), which then yields identical exciton densities for the inner and outer tube.

While the calculation of the exciton densities is straightforward in the case of complete nanotubes, special care had to be taken in the case of isolated inner tubes due to the dissolution of the outer wall and, hence, removal of molecules from the experimentally observable spectral window. Because the monomer absorption is strongly blue-shifted with respect to the nanotube absorption ($\lambda_{\text{max}} \approx 520$ nm, Supplementary Figure 1), the second bracketed factor in Supplementary Equation 1 already accounts for the reduced spectral overlap. Therefore, only the number of molecules that remains embedded in the inner tube has to be estimated for which we use two different ways, i.e., (1) via the optical density (OD) of the monomer absorption spectrum and (2) directly via the absorption of the inner tubes.

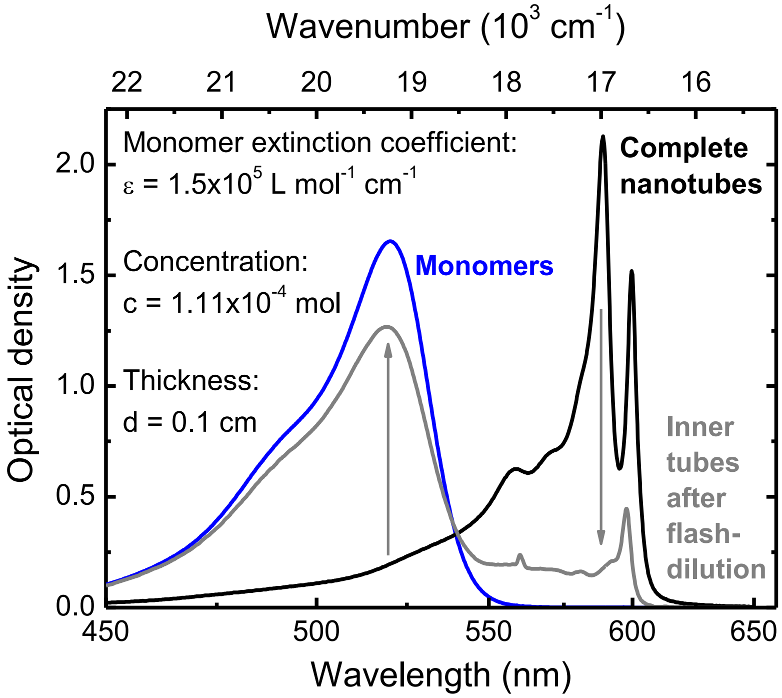

Supplementary Figure 1. Absorption spectra of C8S3 monomers (blue), complete nanotubes (black) and flash-diluted inner tubes (gray). The arrows indicate the main spectral changes upon flash-dilution, i.e., dissolution of the outer layer. The peak monomer extinction coefficient is specified by the supplier (FEW chemicals, Wolfen, Germany) as $\epsilon = 1.5 \times 10^5\ \mathrm{M}^{-1}\ \mathrm{cm}^{-1}$. In the experiment, the molar concentration of the sample was $c = 1.11 \times 10^{-4}$ M and the cuvette thickness $d = 0.1$ cm.

Starting with the former, an upper estimate for the monomer absorption is found by assuming that all molecules ($c = 1.11 \times 10^{-4}$ M) are dissolved. In that case the expected optical density amounts to $\mathrm{OD_{max}} = \epsilon\, c\, d = 1.66$, where ϵ is the extinction coefficient of dissolved C8S3 molecules. In the experiment, however, an optical density of only $\mathrm{OD_{exp}} = 1.27$ is observed upon flash-dilution. The ratio of these optical densities of $\mathrm{OD_{exp}}/\mathrm{OD_{max}} = 0.77$ thus indicates that 77% of the molecules were dissolved due to flash-dilution. This, in turn, leaves a concentration of $c = 2.6 \times 10^{-5}$ M for the molecules that still reside in a nanotube after flash-dilution.

The second estimate for the molar concentration is based on the fact that about 60% and 40% of the molecules reside in the outer and inner layer, respectively[2]. Therefore, in case of perfect flash-dilution, where all inner tubes stay intact, one expects a monomer concentration of $c = 6.67 \times 10^{-5}$ M, which would lead to an $\mathrm{OD} \approx 1$ at 520 nm. This has to be considered as a lower limit of the monomer absorption and clearly underestimates this contribution under the experimental conditions, where the discrepancy arises from the complete dissolution of nanotubes. In fact, the main absorption peak of the inner tubes (~599 nm) decreases by a factor of ~0.7, which indicates that only ~30% of the nanotubes survive flash-dilution. Including this additional rescaling factor, one finds $c = 1.33 \times 10^{-5}$ M, which is in good agreement with the estimate from the monomer absorption. For the calculation of the exciton density, we use the average of both concentrations: $c = (1.94 \pm 0.64) \times 10^{-5}$ M.

The low-energy main transition of the isolated inner tubes appears blue-shifted by ~50 cm^{-1} relative to the corresponding transition in case of complete nanotubes, which is consistent with earlier findings from bulk flash-dilution experiments reported in literature[2]. It has previously been shown that the nanotubes' absorption spectrum depends critically on the tube radius[3] so that we hypothesize that stripping of the outer layer leads to slight inflation of the inner tubes' radius, which in turn causes the blue-shift.

Supplementary Note 2: C8S3 Monomers Signal via One- and Two-Photon Absorption

In this section we verify that the 2D spectra of the inner tube do not contain any contribution from dissolved C8S3 monomers left after flash-dilution. For that, we examine the spectral regions where signals from the monomers are expected following absorption of one photon in the small overlap region between monomer absorption and excitation spectrum (Figure 1b in the main text), or following two-photon absorption via the second electronic excited or high-lying vibronic states. Based on the photoluminescence (PL) emission spectrum of dissolved C8S3 monomers (Supplementary Figure 2a; red line) any signal originating from the monomers is expected to be the strongest in the spectral region between 17000 cm^{-1} and 19000 cm^{-1} along the detection axis (marked by vertical dashed lines and boxes in Supplementary Figure 2a and c, respectively) following ultrafast internal relaxation from the high lying states (Supplementary Figure 2b). The latter would lead to a population of the first electronic excited state of C8S3 monomers, which could then be probed as either a ground-state bleach (GSB) or stimulated emission (SE) signal.

Along the excitation axis, absorption of a single photon in the small overlap region of the excitation spectrum and monomer absorption spectrum would give rise to a signal around ~18200 cm^{-1}, whereas for two-photon absorption we consider the full bandwidth supported by the excitation pulse spectrum, i.e., from 32000 cm^{-1} to 36000 cm^{-1}. In Supplementary Figure 2b this is schematically depicted as 2 × ~590nm, which marks the center of the excitation pulse spectrum, although theoretically any frequency combination may contribute a signal.

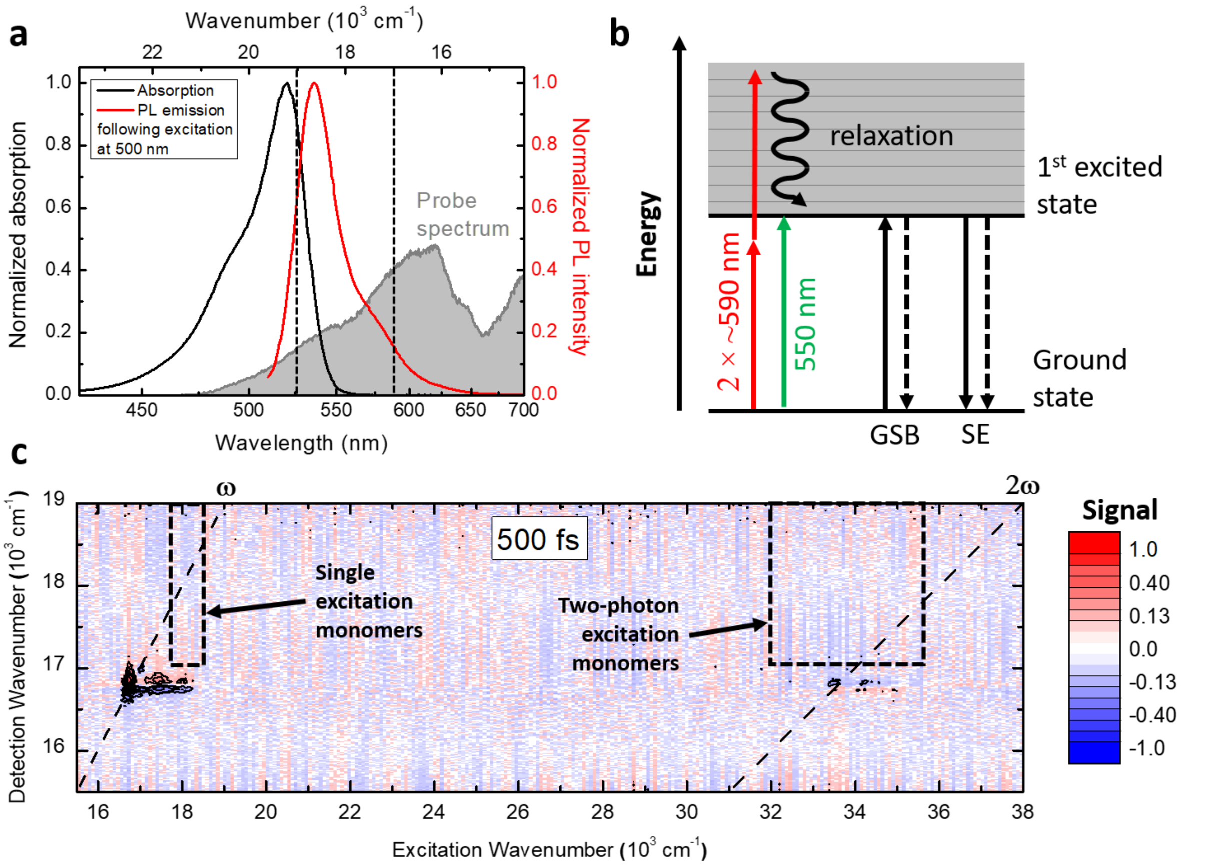

Supplementary Figure 2. (**a**) Normalized linear absorption (black line) and photoluminescence (red line) spectra of C8S3 monomers dissolved in MeOH. A typical probe spectrum used in 2D experiments is shown in gray for comparison. Dashed boxes highlight the detection area relevant for C8S3 monomers. (**b**) Jablonski diagram for the excitation of C8S3 monomers via single photon at the red edge of C8S3 absorption (~550 nm; green arrow) or two-photon absorption at the peak of the excitation spectrum (2 × ~590 nm; red arrows). The latter is followed by ultrafast relaxation (wiggly arrow). Population in the first excited state is then probed via GSB or SE processes as indicated by the arrows. (**c**) 2D absorptive and EEI2D spectrum for isolated inner tubes for a waiting time of 500 fs under the highest excitation density, i.e., one exciton per ~20 inner-tube molecules. The spectrum was normalized to the maximum absolute signal. The signal amplitude is depicted on a color scale with increments at 0.83, 0.57, 0.4, 0.27. 0.19, 0.13, 0.08, 0.05, 0.03, and 0.01 to ensure the visibility of low amplitude signals. Contour lines were drawn as specified in the color bar. The respective spectral regions of interest where a signal from C8S3 monomers is expected, are marked by boxes (black dashed) and labelled accordingly.

Examination of these two spectral regions of interest for the monomer response in EEI2D experiments reveals that in neither case we observe any distinct signal originating from the monomers at the background of low-amplitude noise. Since any monomer signal would be strongest in these spectral windows, we conclude that the spectral region along the detection axis around ~600 nm (~16670 cm^{-1}) which is relevant to the inner tube response, is free of any monomer signal.

We further support this observation by estimating the exciton density of monomers. The small overlap region of the low-energy tail of the monomer absorption spectrum and the laser excitation spectrum at around 550 nm (Figure 1b in the main text) could lead to weak excitation of C8S3 monomers. For the calculation of the monomer exciton density, we use an (average) monomer concentration of $c = 9.06 \times 10^{-5}$ M after flash-dilution as estimated in the preceding section and find an exciton density of one excitation per ~3300 C8S3 monomers under the highest excitation fluence in the experiment. Simultaneously, the exciton density for the inner tubes in the same experiment is about one exciton per ~20 molecules, which is a factor of ~165 higher than for the monomers. Therefore, we conclude that excitation of monomers via absorption of one photon in the low-energy tail of the monomer absorption is negligible given the small spectral overlap with the excitation spectrum. The fact that the two-photon absorption cross section of C8S3 monomers is not known prevents such an estimate for two-photon absorption. However, as the two-photon excitation proceeds via a non-resonant state, its cross-section is expected to be even lower. These estimates support the absence of the monomer signals in the absorptive and EEI2D spectra.

Supplementary Note 3: Integration of the Absorptive and EEI Signals

In order to retrieve the absorptive and EEI transients for isolated inner tubes as well as complete nanotubes (Figures 3, Figure 4 and Figure 5 in the main text), the 2D spectra were integrated in the rectangular regions of interest as depicted in Figure 2 in the main text. The exact integration intervals are specified in Supplementary Table 1.

Supplementary Table 1. Integration intervals for the absorptive and EEI signal transients of isolated inner and complete nanotubes.

		Absorptive signal	EEI signal
Isolated inner tubes	Inner tube diagonal peak	Exc. [16625 cm^{-1}, 16875 cm^{-1}]<br>Det. [16680 cm^{-1}, 16780 cm^{-1}]	Exc. [33375 cm^{-1}, 33625 cm^{-1}]<br>Det. [16680 cm^{-1}, 16780 cm^{-1}]
Complete nanotubes	Outer tube diagonal peak	Exc. [16925 cm^{-1}, 17175 cm^{-1}]<br>Det. [16900 cm^{-1}, 17000 cm^{-1}]<br>(or [17000 cm^{-1}, 17100 cm^{-1}])	Exc. [33975 cm^{-1}, 34225 cm^{-1}]<br>Det. [16900 cm^{-1}, 17000 cm^{-1}]<br>(or [16950 cm^{-1}, 17050 cm^{-1}])
	Inner tube diagonal peak	Exc. [16585 cm^{-1}, 16835 cm^{-1}]<br>Det. [16600 cm^{-1}, 16700 cm^{-1}]<br>(or [16650 cm^{-1}, 16750 cm^{-1}])	Exc. [33295 cm^{-1}, 33545 cm^{-1}]<br>Det. [16600 cm^{-1}, 16700 cm^{-1}]<br>(or [16650 cm^{-1}, 16750 cm^{-1}])
	Cross peak (outer → inner)	Exc. [16925 cm^{-1}, 17175 cm^{-1}]<br>Det. [16600 cm^{-1}, 16700 cm^{-1}]<br>(or [16650 cm^{-1}, 16750 cm^{-1}])	Exc. [33975 cm^{-1}, 34225 cm^{-1}]<br>Det. [16600 cm^{-1}, 16700 cm^{-1}]<br>(or [16650 cm^{-1}, 16750 cm^{-1}])

In practice, vertical slices of the 2D spectra were averaged over 250 cm^{-1} (corresponding to three data points) along the excitation axis. Next, the baseline was subtracted from these vertical slices and the respective signal of interest was averaged along the detection axis over 100 cm^{-1} (corresponding to 10 data points). Due to the increased number of features in the absorptive 2D and EEI2D spectra in the case of complete nanotubes, individual contributions from GSB/SE and ESA with opposite signs are more likely to spectrally overlap and, hence, partially compensate

each other. At the highest exciton density and, hence, the strongest signals we found this partial compensation to lead to peak shifts, which we accounted for by slightly adjusting the integration area (specified in parenthesis in Supplementary Table 1) in order to avoid simultaneous integration over negative and positive signals.

One of the dominant sources of uncertainty of the extracted signal amplitudes were fluctuations of the background due to unsuppressed scattering of the pump and probe pulses. We determine the standard error of these background fluctuations during each measurement (i.e., at a given exciton density) for the respective spectral regions of interest for the absorptive and EEI signals. The same excitation frequency limits (Supplementary Table 1) are used as before from which the background signal is extracted for each waiting time in the spectral interval from 16000 cm^{-1} to 16200 cm^{-1} along the detection axis. The error bars are identical for all waiting times within the same scan, but may be slightly different for the absorptive and EEI signals.

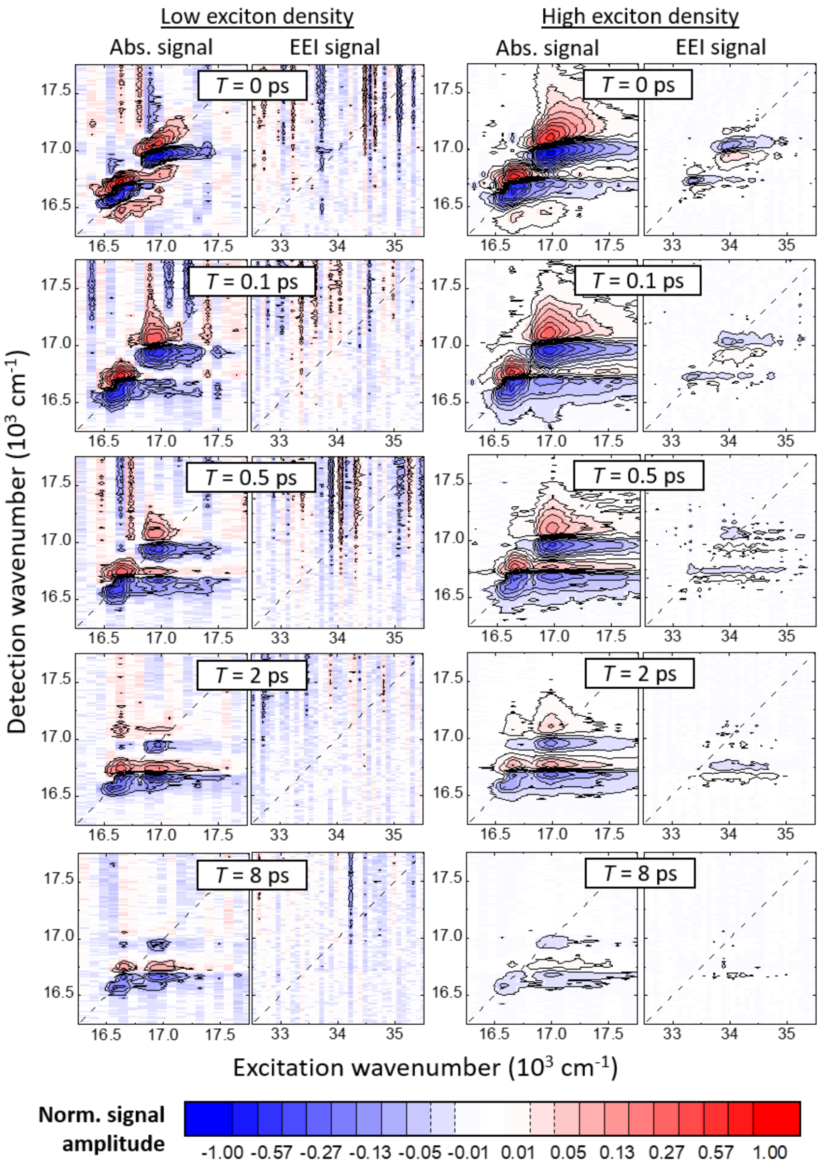

Supplementary Figure 3. Absorptive 2D and EEI2D spectra of complete nanotubes recorded at low (1 exciton per ~600 molecules; left column) and high (1 exciton per ~60 molecules; right column) exciton densities for a range of waiting times. All shown spectra are normalized to the maximum absolute amplitude of the respective absorptive signal at zero waiting time, which preserves the relative scaling between the absorptive and EEI signals. The signal amplitude is depicted on a color scale (between -1 and +1) with increments at 0.83, 0.57, 0.4, 0.27. 0.19, 0.13, 0.08, 0.05, 0.03, and 0.01 to ensure visibility of all peaks at all waiting times. For the spectra at high exciton density all contour lines are drawn, whereas for the low exciton density spectra the contour lines of the lowest levels are omitted as indicated on the color bar (dashed lines are not used for low exciton density).

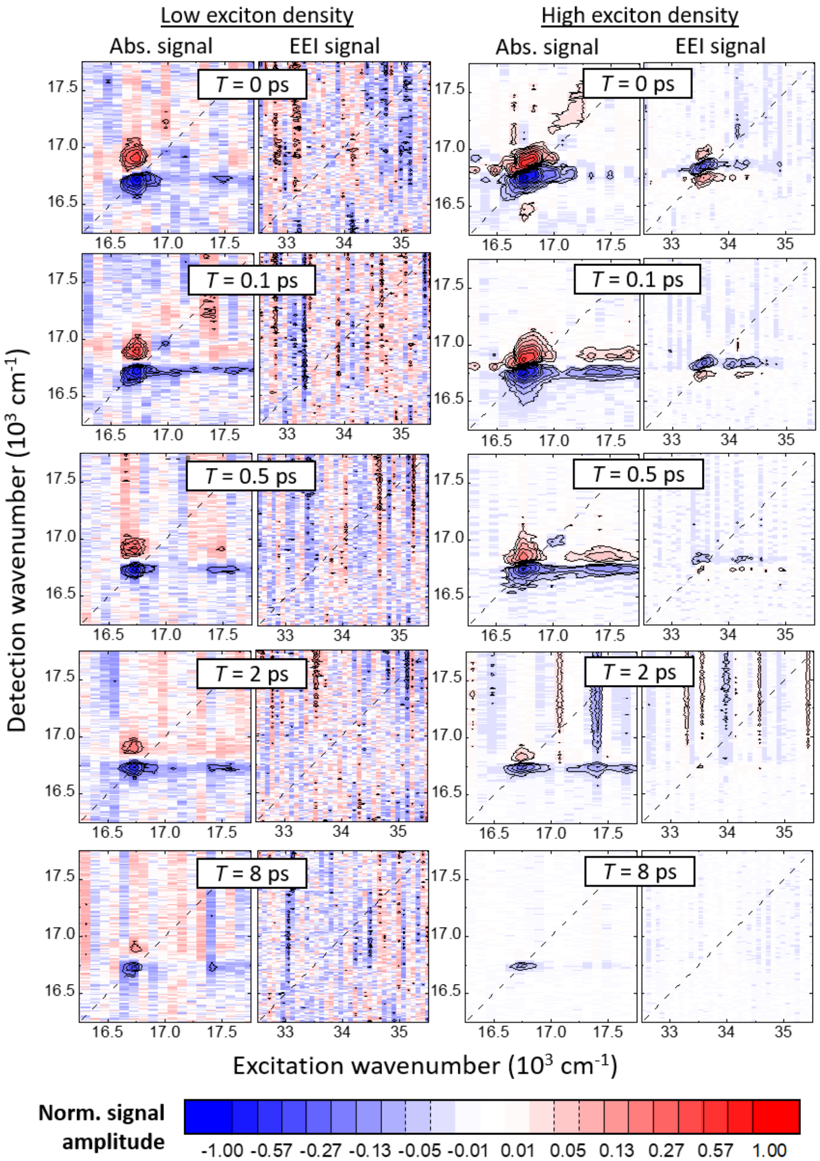

Supplementary Figure 4. Absorptive 2D and EEI2D spectra of isolated inner tubes recorded at low (1 exciton per ~400 molecules; left column) and high (1 exciton per ~20 molecules; right column) exciton densities for a range of waiting times. All shown spectra are normalized to the maximum absolute amplitude of the respective absorptive signal at zero waiting time, which preserves the relative scaling between the absorptive and EEI signals. The signal amplitude is depicted on a color scale (between -1 and +1) with increments at 0.83, 0.57, 0.4, 0.27. 0.19, 0.13, 0.08, 0.05, 0.03, and 0.01 to ensure visibility of all peaks at all waiting times. The drawn contour lines are indicated on the color bar (dashed lines are not used for low exciton density).

Supplementary Note 4: Absorptive and EEI Cross Peaks from Intra-Band Relaxation

In the case of isolated inner tubes, weak cross peaks can be identified in the absorptive 2D and EEI2D spectra at the detection frequency of the inner tubes (ω_{inner}) at higher excitation frequencies. The appearance of these cross peaks is linked to one of the blue-shifted transitions of the nanotube absorption spectrum (Figure 1b in the main text), which originates from the complex molecular packing with two molecules per unit cell[2]. In fact, each molecule in the unit cell gives rise to two excitonic transitions, one of which is polarized parallel and the other orthogonal to the nanotubes' long axis[4]. As a result, the absorption spectrum of the inner tubes comprises a total of four transitions, out of which only the parallel polarized transitions at ~16750 cm^{-1} and ~17500 cm^{-1} are relevant for 2D spectroscopy due to polarization-selective excitation. The latter was facilitated by the polarization of the excitation pulses set parallel to the sample flow along which the nanotubes preferentially align due to their large aspect ratio.

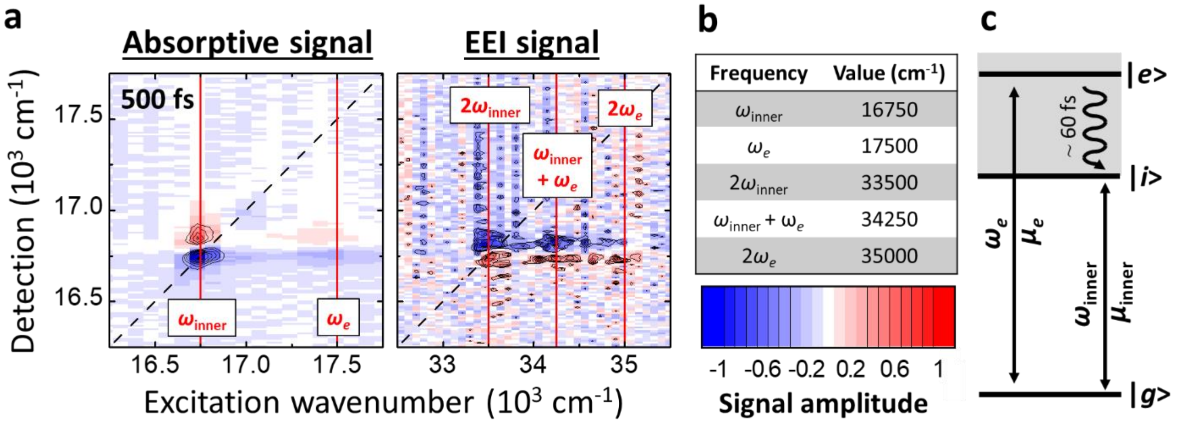

Supplementary Figure 5. Absorptive and EEI cross peak for isolated inner tubes. (**a**) Representative absorptive 2D (left) and EEI2D (right) spectra recorded at a waiting time of 500 fs for isolated inner tubes at the highest exciton density of one exciton per ~20 molecules. The spectra were normalized to their maximum absolute amplitude. The signal amplitude is depicted on a linear color scale (between -1 and +1) with increments of 0.1. Contour lines are drawn as specified in the color bar. Dashed lines are drawn at $\omega_{excitation} = \omega_{detection}$ and $\omega_{excitation} = 2\omega_{detection}$ for absorptive 2D and EEI2D spectra, respectively. (**b**) Summary of the relevant excitation frequencies of optical transitions for isolated inner tubes for absorptive and EEI signals. These frequencies are shown as vertical red lines in the 2D spectra. (**c**) Level diagram of isolated inner tubes with the intra-band exciton state ($|e\rangle$) explicitly drawn. Optical transitions are depicted as vertical arrows with the corresponding frequencies and transition dipole moment indicated. Intra-band exciton relaxation is shown as a wiggly arrow.

The states corresponding to the strong transition at ω_{inner} ~16750 cm^{-1} are situated at the bottom of the exciton band, i.e., the super-radiant states[5], for which an extensive analysis is presented in the main part of the paper. In contrast, the high-frequency transition corresponds to states that lie deep within the exciton band (Supplementary Figure 5c), which we denote as $|e\rangle$ with the corresponding frequency ω_e and transition dipole moment μ_e. Excitation of this transition is followed by ultrafast intra-band relaxation on a sub-100 fs timescale[6], which leads to additional population of the bottom states of the exciton band encoded in a rapidly in-growing

cross peak in the absorptive 2D spectra ($\omega_e \rightarrow \omega_{inner}$). Note that in our experiments the corresponding diagonal peak could hardly be detected because of its short-lived nature and sparse sampling of the waiting time. However, previously published transient absorption (TA) data revealed a decay time as short as ~60 fs for this transition[7]. An additional complication in measuring the diagonal peak arises from the fact that its amplitude scales with the already small dipole moment ($|\mu_e|^4$), whereas the cross peak involves the stronger transition dipole moment of the inner tube ($|\mu_e|^2|\mu_{inner}|^2$) and is therefore easier to detect. In comparison, the same cross peak ($\omega_e \rightarrow \omega_{inner}$) is present in the absorptive 2D spectra of complete nanotubes, but only visible as peak elongations towards higher excitation frequencies (Figure 2b in the main text), as they partially overlap with the much stronger cross peak due to the outer layer.

Following ultrafast intra-band relaxation, excitons can further diffuse and eventually undergo exciton–exciton annihilation, which is reflected in the emergence of cross peaks in the EEI2D spectra (Supplementary Figure 5a). Specifically, the strongest cross peak is observed at a detection frequency ω_{inner} and an excitation frequency of 34250 cm^{-1} (marked by the center vertical line at $\omega_{inner} + \omega_e$ in Supplementary Figure 5a), which corresponds to the sum of the contributing excitation frequencies, i.e., (16750 + 17500) cm^{-1} (see table in Supplementary Figure 5b). In comparison, the other vertical lines refer to the fundamental transitions at excitation frequencies of $2\omega_{inner}$ and $2\omega_e$. The EEI cross peak ($\omega_{inner} + \omega_e \rightarrow \omega_{inner}$) encodes the mutual interaction of excitons one of which was directly excited at the bottom of the exciton band, whereas the other one underwent intra-band relaxation. Note that for complete nanotubes the spectral region around the detection frequency ω_{inner} is dominated by the EEI cross peak at double the excitation frequency of the outer tube ($2\omega_{outer}$ = 34000 cm^{-1}).

Supplementary Note 5: Double-Sided Feynman Diagrams for the EEI Signals

5.1. EEI Diagonal Peaks

In this section we present the double-sided Feynman diagrams that contribute to the EEI signal of the diagonal peak of the isolated inner tubes (Supplementary Figure 6) and the diagonal peak of the outer tube (Supplementary Figure 7). For conciseness, only the rephasing diagrams as determined by their phase matching condition, i.e., $k_{\text{signal}} = -2k_{pu} + 2k_{pu} + k_{pr}$, are shown[8], where k_{pu} is the wavevector of the pump beam, k_{pr} the wavevector of the probe beam, and detection occurs along direction k_{signal}. The non-rephasing diagrams can be derived by considering diagrams that emit a signal field in the phase-matched direction of $+2k_{pu} - 2k_{pu} + k_{pr}$. However, a rigorous mathematical analysis, where all signals are computed in the framework of the response function theory and subsequently convoluted with the electric fields of the involved laser pulses is beyond the scope of this work[8,9].

For the EEI diagonal peak of isolated inner tubes we consider diagrams that give rise to a signal at an excitation frequency of $2\omega_{\text{inner}}$ and detection at ω_{inner}. In the diagrams for the isolated inner tubes $|g\rangle$ represents the electronic ground-state, $|i\rangle$ and $|j\rangle$ the one-exciton states of the inner tube, $|ii\rangle$ and $|jj\rangle$ bi-exciton states, and analogously $|jjj\rangle$ for the tri-exciton states (see the level diagram in Supplementary Figure 6. Note that we formally distinguish between the states $|i\rangle$ and $|j\rangle$ (and $|ii\rangle$ and $|jj\rangle$) of the two neighboring excitons to include the fact that the exciton state after the waiting time T is not necessarily identical to the exciton state prepared by the pump pulses.

All diagrams in Supplementary Figure 6 share the general structure that the first four interactions with the pump pulses ($\mp 2k_{pu}$) excite the inner tube followed by the probe pulse ($+k_{pr}$) interacting with the same tube. During the waiting time *T*, exciton–exciton annihilation (EEA) can occur (Supplementary Figure 6, right column; highlighted in orange), if the system is in a bi-exciton population state. As shown in literature the EEI signal is then dominated by EEA[1,10].

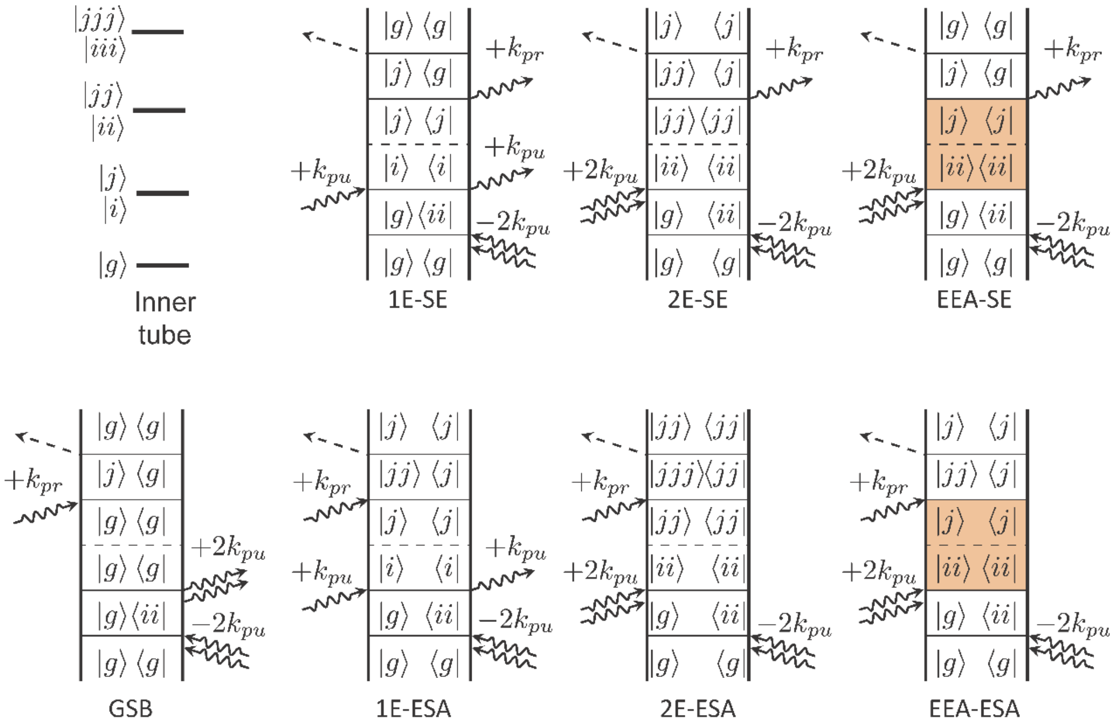

Supplementary Figure 6. Rephasing double-sided Feynman diagrams, which contribute to the EEI diagonal peak of isolated inner tubes ($2\omega_{\text{inner}} \rightarrow \omega_{\text{inner}}$). The level diagram for the isolated inner tubes is shown in the upper left corner. In the diagrams time flows from the bottom to the top during which the interactions with the laser pulses are indicated by arrows. The dashed line indicates propagation during the waiting time *T*. The double interaction with each of the two pump pulses can create a population of the ground state, a one-exciton (1E) state or a bi-exciton (2E) state, which are subsequently probed by GSB ($|g\rangle \rightarrow |j\rangle$), SE ($|j\rangle \rightarrow |g\rangle$ or $|jj\rangle \rightarrow |j\rangle$) or ESA ($|j\rangle \rightarrow |jj\rangle$ or $|jj\rangle \rightarrow |jjj\rangle$). The process of exciton–exciton annihilation (EEA) is shaded in orange.

In absence of exciton transfer (ET) between the tubes, the description of the outer tube diagonal peak is identical to the isolated inner tube with exception of the notation of the states. Hence, the former can be obtained by renaming the states according to $|i\rangle \rightarrow |o\rangle$, $|ii\rangle \rightarrow |oo\rangle$, *etc*. Nevertheless, we explicitly include these diagrams in Supplementary Figure 7 here, as they form the basis for the discussion on the EEI cross peak in the next section.

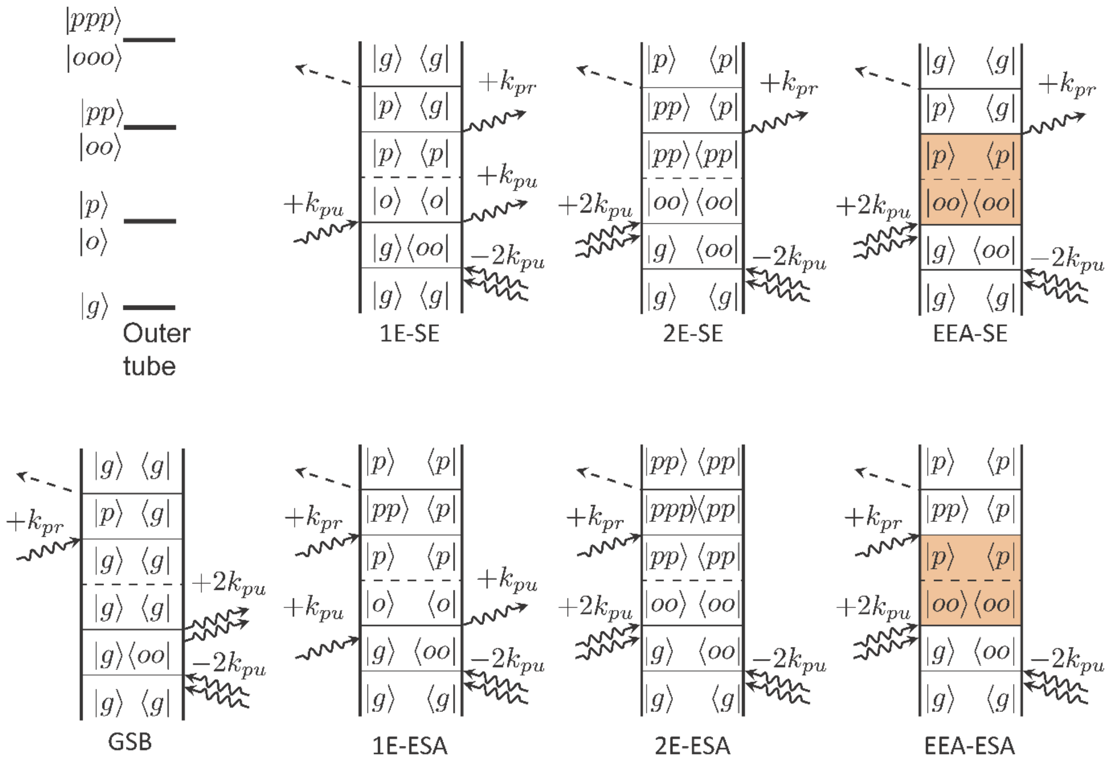

Supplementary Figure 7. Rephasing double-sided Feynman diagrams, which contribute to the EEI diagonal peak of the outer tube. The level diagram for the outer tube assuming that the absence of any inter-tube ET, is shown in the upper left corner.

5.2. EEI Cross Peak

In order to understand the character of the EEI cross peak we discuss the double-sided Feynman diagrams, which contribute to the particular signal at double the excitation frequency of the outer tube with subsequent detection at the frequency of the inner tube, i.e., $2\omega_{\text{outer}} \rightarrow \omega_{\text{inner}}$. At time zero ($T = 0$), where neither exciton transfer (ET) nor EEA occur, only three diagrams contribute to the EEI cross peak signal (Supplementary Figure 8, black box). Here, the first four interactions with the pump pulses excite the outer tube, while the probe pulse interacts with the inner tube. Since the outer and inner tubes can be considered as weakly coupled[2], excitation of the outer tube does not influence the inner tube and *vice versa*. Due to the weak coupling we also exclude the possibility of any inter-tube exciton–exciton annihilation, where two excitons residing on different tubes annihilate directly without any ET event involved. Therefore, the state labelled as $|pi\rangle$ (see level diagram in Supplementary Figure 8) refers to the situation of two independent excitons – one on each tube. Analogously, $|ppi\rangle$ describes the situation of one exciton located on the inner and two excitons located on the outer tube. Due to the different overall signs of the diagrams (Supplementary Figure 8, black box) all different pathways mutually compensate each other and, hence, no EEI cross peak is visible. Analogously, it can be shown that for weakly coupled systems the Feynman diagrams corresponding to the absorptive cross peak ($\omega_{\text{outer}} \rightarrow \omega_{\text{inner}}$) cancel each other at zero waiting time[11].

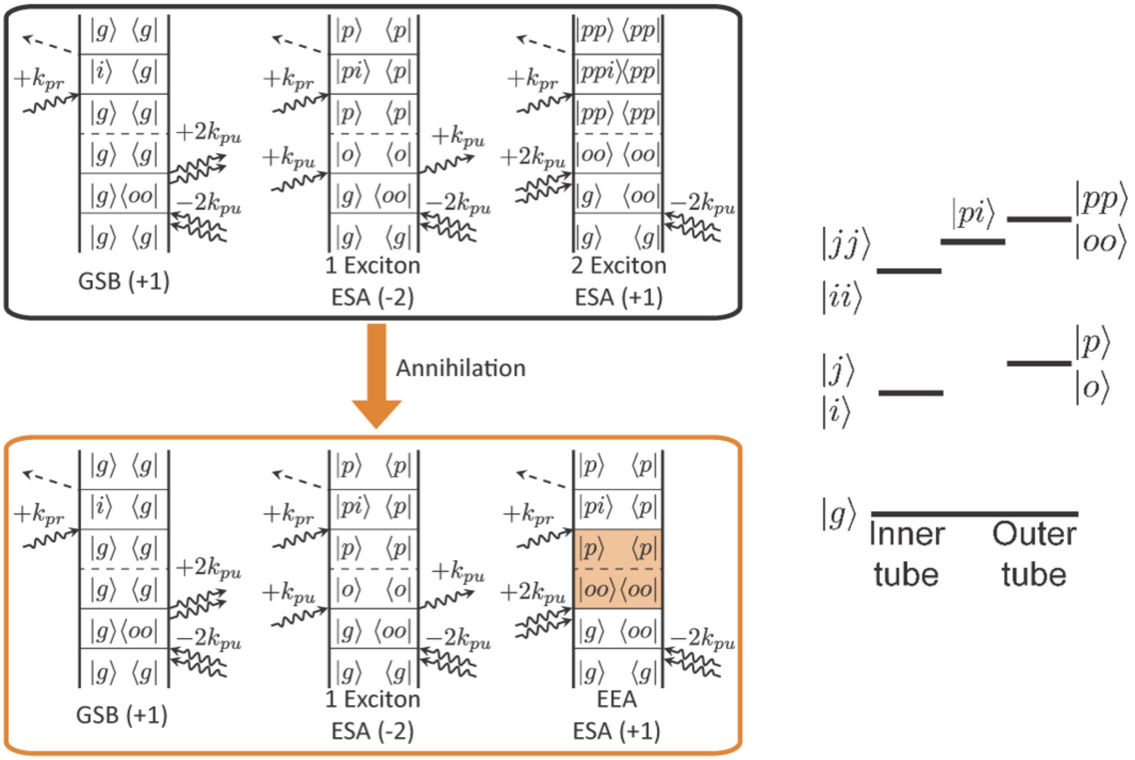

Supplementary Figure 8. Rephasing double-sided Feynman diagrams for the EEI cross peak at zero waiting time. Top panel (black): Pathways without inter-tube exciton transfer (ET) and EEA. Bottom panel (orange): Possible pathways including EEA, but still no ET. The level diagram for complete nanotubes is shown on the right side.

EEA on the outer tube in absence of any ET leads to a modified set of diagrams for the EEI cross peak, where the third diagram is replaced by one that contains EEA (Supplementary Figure 8, orange box; diagram on the right). However, under the premise of no ET and assuming weak coupling, EEA on the outer tube does not influence the exciton dynamics on the inner tube and, thus, does not alter the interaction of the probe pulse with the latter. Therefore, the diagrams at zero waiting time still mutually cancel, as it is the case in upper panel of Supplementary Figure 8 and, thus, no EEI cross peak emerges.

For finite waiting times *T*, inter-tube ET has to be considered explicitly. As a result, the condition that excitons on the outer tube will not influence exciton processes on the inner tube does not hold any longer. The corresponding double-sided Feynman diagrams, which contain both contributions, i.e., ET (shaded in blue) and EEA (shaded in orange), are shown in Supplementary Figure 9. As shown in literature the EEI signal is dominated by pathways that include EEA[1,10], although these diagrams co-exist with a number of diagrams that contain only ET, which formally also give rise to a signal at $2\omega_{\text{outer}} \rightarrow \omega_{\text{inner}}$. The fact that the EEI cross peak dynamics in experiment (see Figure 5 in the main text) exhibit a dependence on the excitation intensity corroborates the fact that the diagrams containing both ET and EEA are most relevant to the EEI cross peak, as for the diagrams with ET alone no intensity dependence is expected.

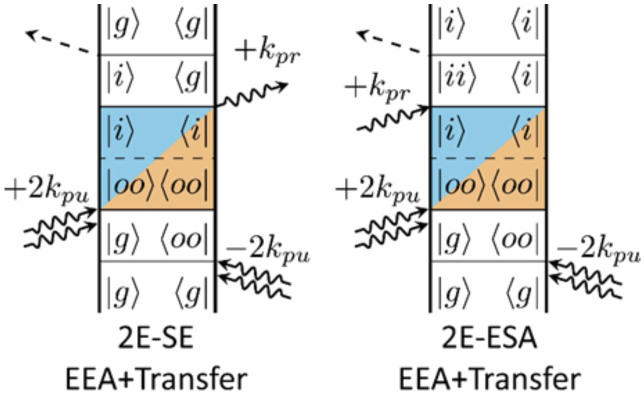

Supplementary Figure 9. Rephasing double-sided Feynman diagrams which contribute to the cross peak of the EEI signal. Additional to EEA (orange) population transfer is included (blue).

5.3. Seventh-Order Signals

As discussed in the main text, higher-order, namely at least seventh order, effects occur at high exciton densities, which were indeed observed experimentally at three times of the fundamental frequency (Supplementary Note 7). Although the seventh-order signal can be glimpsed spectroscopically isolated at triple the fundamental frequency, it also contributes to the (fifth-order) EEI signal at twice the fundamental frequency. This occurs in a similar fashion as the (fifth-order) EEI signal contributes to the (third-order) absorptive signal, as the transient exciton dynamics accelerate in presence of exciton–exciton annihilation. Two diagrams that demonstrate the effect of the seventh-order on the EEI signal of the diagonal peak of the outer tube (left diagram; $2\omega_{\text{outer}} \rightarrow \omega_{\text{outer}}$) and the cross peak (right diagram; $2\omega_{\text{outer}} \rightarrow \omega_{\text{inner}}$) are shown in Supplementary Figure 10a. Two exemplary diagrams for the diagonal peak and the cross peak at three times the excitation frequency $3\omega_{\text{outer}}$ are shown in Supplementary Figure 10b.

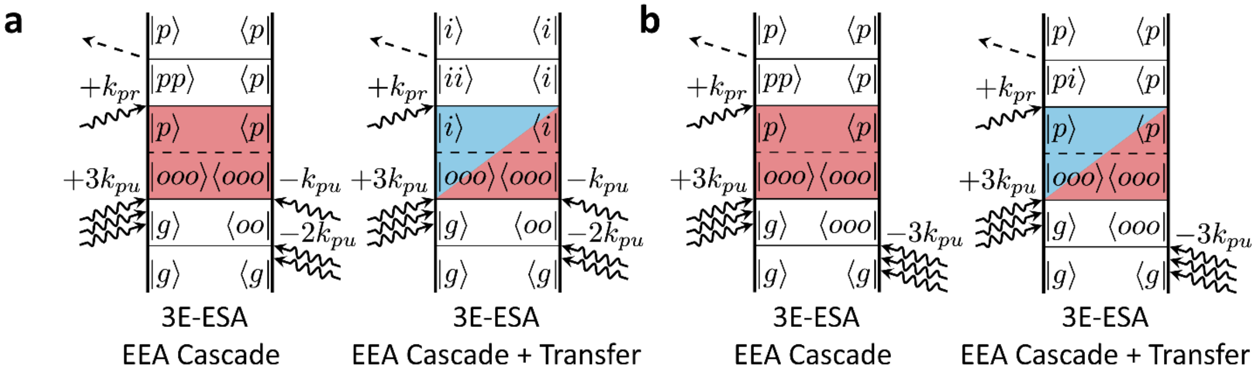

Supplementary Figure 10. (**a**) Rephasing double-sided Feynman diagrams of the seventh-order signal which give rise to a signal at an excitation frequency of $2\omega_{\text{outer}}$ and detection at ω_{outer} or ω_{inner} following ET. EEA cascades are shown in dark red, while ET from the outer tube to the inner tube is shaded in blue. (**b**) Rephasing double-sided Feynman diagrams for the signal at $3\omega_{\text{outer}}$.

Possible contributions to the seventh-order signal include a sequential cascade of annihilations, in which excitons participate in multiple EEA events during the waiting time *T*. For example, after a bi-exciton state relaxes to a one-exciton state, it is excited one more time to the bi-exciton state that subsequently relaxes. However, such processes are not very likely in our experiments because of the short (~15 fs) pulse duration, the low intensity of the probe pulse and a single-pump-beam geometry, which prevents the second excitation to occur with a photon from the same pump pulse.

Supplementary Note 6: Monte-Carlo Simulations

6.1. Simulation Grid

The molecular grid for Monte-Carlo (MC) simulations was set up to match the size known from cryo-TEM measurements[2,12,13] and previously published theoretical models[2]. The boundary conditions for the grid are given by the radii and the molecular surface densities of both tubes. For simplicity we assume identical square grids for the inner and outer tube with a single molecule on each grid site, although more sophisticated models for the molecular packing have been proposed including brickwork models[14–16] and extended herringbone models[2,3]. Here, we use the same model parameters as in ref. 2: $R_{\mathrm{outer}} = 6.465$ nm and $R_{\mathrm{inner}} = 3.551$ nm for the radii, $N_{\mathrm{outer}} = 14260$ and $N_{\mathrm{inner}} = 7992$ for the number of molecules in each tube and a total tube length of $L \approx 197$ nm. From these values the molecular surface densities are calculated as $\rho = N/2\pi RL$. In order to construct the square grid for MC simulations we use the average molecular surface density of both layers ~1.81 molecules nm^{-2}, from which the lattice constant is calculated as $a = \sqrt{1/\rho} \approx 0.74$ nm. The number of molecules on the circumference then follows as $N_c = 2\pi R/a$. A summary of the relevant parameters for the molecular grid is given in Supplementary Table 2.

Supplementary Table 2. Summary of parameters for the molecular grid for MC simulations. The chemical structure of C8S3 is shown in the right column with dimensions indicated. Note that the latter refers to the situation with the octyl side-groups fully extended.

Quantity	Inner layer	Outer layer	Chemical structure
Radius (ref. 2)	3.551 nm	6.465 nm	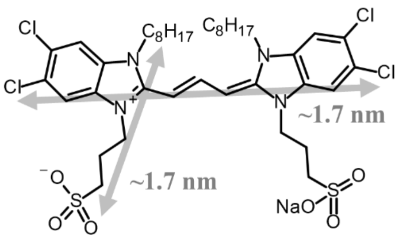
Circumference	~22.3 nm	~40.6 nm	
Molecular surface density	~1.84 molecules nm^{-2}	~1.77 molecules nm^{-2}	
Lattice constant	~0.74 nm		
Unit cell area	~0.55 nm^2		
Number of molecules on the circumference	~30	~55	

At first glance the value for the lattice constant disagrees with the molecular geometry, as the size of the molecule exceeds this length (see Supplementary Table 2 for chemical structure). However, it is important to realize that assuming a simple square grid for the molecular packing yields a single effective lattice constant, which averages the actual separation between individual molecules in different directions for more sophisticated packing motifs. In reality, the molecules are expected to stack in one direction with their chromophores closely aligned at distances on the order of 0.4 nm (as reported for a structurally similar molecule[17]), while the molecular separation in the “lateral” direction would roughly correspond to the molecular size of 1.7 nm. Combined this yields a unit cell area of 1.7 nm × 0.4 nm = 0.68 nm^2, which agrees well with the unit cell area derived from the square grid. The remaining molecular dimension (~1.7 nm) sticks out perpendicular from the planes considered here and contributes to the wall thickness of the double-walled assembly. According to the inner and outer radii the thickness amounts to ~3 nm, which is in good agreement with two times the molecular size in this direction.

6.2. Extraction of the Absorptive and EEI Signal

Supplementary Table 3 summarizes the criteria after which the absorptive and EEI signals for spectra were extracted from the MC simulations. In order to obtain the amplitude of a specific peak at time *t* in the simulations only excitons fulfilling the below criteria were counted.

Supplementary Table 3. Prerequisites for extraction of the absorptive and EEI signals from MC simulations.

Signal	Peak type	Origin	Annihilated?	Position at time t	Freq. (exc.→det.)
Absorptive	diagonal peak	inner	-	inner	$\omega_{inner} \rightarrow \omega_{inner}$
	diagonal peak	outer	-	outer	$\omega_{outer} \rightarrow \omega_{outer}$
	cross peak	outer	-	inner	$\omega_{outer} \rightarrow \omega_{inner}$
EEI	diagonal peak	inner<br>inner	yes	inner	$2\omega_{inner} \rightarrow \omega_{inner}$
	diagonal peak	outer<br>outer	yes	outer	$2\omega_{outer} \rightarrow \omega_{outer}$
	cross peak	outer<br>outer	yes	inner	$2\omega_{outer} \rightarrow \omega_{inner}$

6.3. Definition of the Annihilation Radius

We use the annihilation radius as a quantity to characterize the distance dependence of the interactions of two approaching excitons that ultimately results in annihilation of one of the excitons. If we assume a Förster-type exchange for an exciton–exciton annihilation event[18–20], the probability of the event scales with distance R as $(1 + R^6/R_0^6)^{-1}$, where R_0 is the Förster radius. In order to ease the computations, we approximate this dependence by a step function: the probability of annihilation within R_0 is unity, otherwise it is null (Supplementary Figure 11). In

other words, within the cut-off distance R_0 the annihilation event outcompetes any other relevant rate in the system (e.g., the exciton decay rate and the exciton diffusion rate).

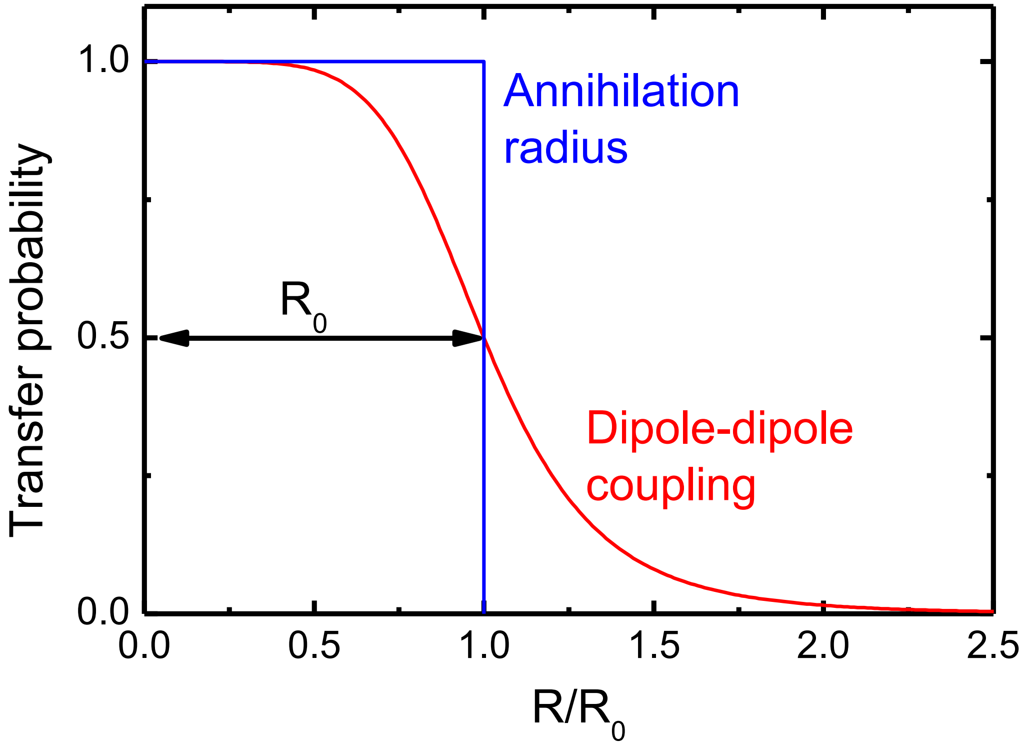

Supplementary Figure 11. Distance dependence of the Förster-type transfer probability (red) and its approximation with a step function (blue; here denoted as R_0).

6.4. Exciton displacement

In order to compute the mean (square) exciton displacement Monte-Carlo simulations were conducted in an annihilation-free setting, i.e., with EEA switched off, and at a low exciton density. The latter was important to not hinder the exciton motion by having too many occupied sites. All excitons were labelled with their initial [X_i, Y_i, Z_i] and final [X_f, Y_f, Z_f] position on the

grid, where the *X* and *Y* coordinates refer to sites along and across the molecular grid, respectively. The *Z* coordinate encodes whether an exciton resides on the inner or outer layer for which *Z* can take values of 0 or 1. As inter-layer exciton transfer is constrained to occur vertically, i.e., between corresponding sites on the inner and outer layer, the *Z*-coordinate can be neglected for the calculation of the (square) displacement. Instead, the displacement x_n and square displacement x_n^2 of the nth exciton are computed via:

$$x_n = a\sqrt{[(X_{\text{f}} - X_{\text{i}})^2 + (Y_{\text{f}} - Y_{\text{i}})^2]}, \quad (2)$$

$$x_n^2 = a^2[(X_{\text{f}} - X_{\text{i}})^2 + (Y_{\text{f}} - Y_{\text{i}})^2]. \quad (3)$$

Here, a is the lattice constant (Supplementary Note 6.1). The histograms for the displacement and square displacements for isolated inner tubes and complete nanotubes are shown in Supplementary Figure 12. In the simulations of the isolated inner tubes and complete nanotubes we find mean square exciton displacements ($< x^2 >$) of 1282 nm^2 and 722 nm^2, respectively, which translate into 2319 molecules and 1307 molecules assuming a molecular surface density of 1.81 molecules nm^{-2}. These values differ despite identical exciton hopping rates due to different one-exciton lifetimes, which permits excitons to diffuse longer (and farther) in the case of isolated inner tubes. From the mean square displacement the diffusion constants are calculated ($< x^2 > = 4D_{2D}\tau$)[21]. Note that this diffusion constant refers to the situation of isotropic exciton transport based on the underlying (simplified) molecular square grid, where identical exciton hopping rates in all directions are assumed.

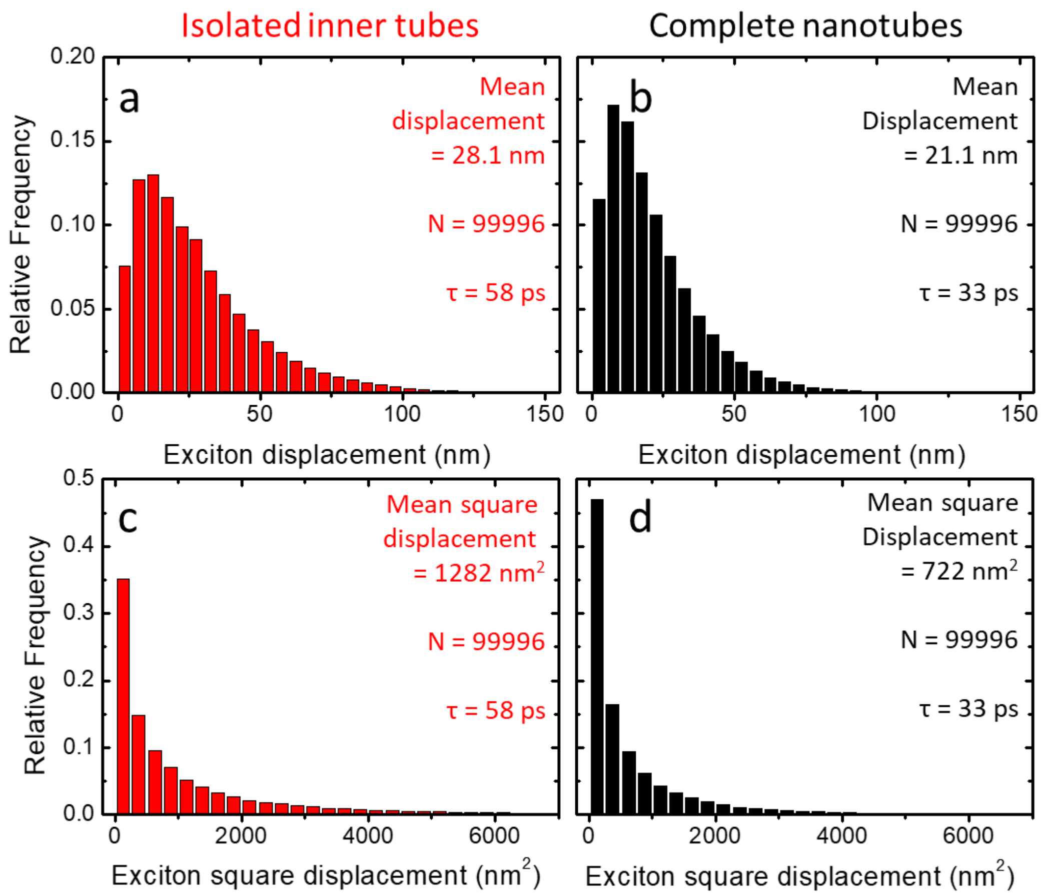

Supplementary Figure 12. Exciton (square) displacement in Monte-Carlo simulations. (**a**) and (**b**) histograms for the exciton displacement, and (**c**) and (**d**) square exciton displacement obtained from Monte-Carlo simulations in case of isolated inner tubes (left, red) and complete nanotubes (right, black). For the histograms, the binning width was set to 5 nm in the case of exciton displacement, and 250 nm^2 for the exciton square displacement. The inset states the mean (square) exciton displacement, number of excitons the statistics are based on and the one-exciton lifetime.

6.5. Exciton–Exciton Annihilation Statistics: Isolated Inner Tubes

In the Monte-Carlo simulations the path of each individual exciton is recorded, during which it could either naturally decay due to its finite lifetime or undergo EEA with another exciton. In that case, one of the excitons is deleted, while the surviving exciton can continue to diffuse and engage in additional EEA events. For the latter, the accumulated number of participations in EEA events was recorded until and including an exciton's own relaxation. As the excitons are not constrained from participation in multiple EEA events, this value may exceed one.

The nature of EEA in which one exciton is destroyed imposes a lower limit on the share of excitons for each number of EEA events. For example, in the extreme case of complete annihilation of all excitons, 50% of the excitons can accumulate a maximum of one annihilation event, 25% of the excitons a maximum of two annihilation events, *etc.*, as described by the geometric distribution. In contrast, in complete absence of exciton–exciton annihilation, excitons decay only according to their lifetime and, thus, accumulate no annihilation events. These two limiting cases dictate the lower and upper bound for the mean number of EEA participations ($< N_{\mathrm{ann}} >$) between 0 for the annihilation free case and 2 for complete annihilation of all excitons. Note that these boundaries only hold for a closed system, i.e., the system does not receive any additional excitons from an external source, as for example via exciton transfer from the outer tube.

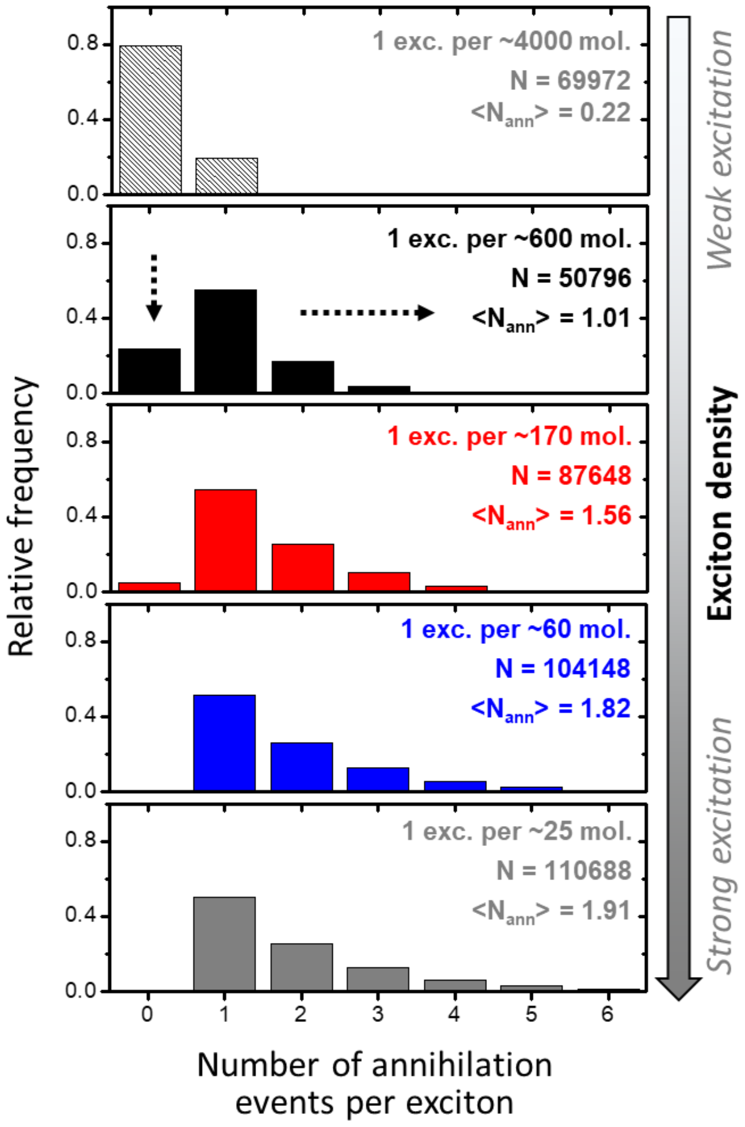

Supplementary Figure 13. Histograms for the number of accumulated annihilation events per exciton in the Monte-Carlo simulations of isolated inner tubes at different exciton densities (shown in the inset of each panel). The inset also specifies the number of excitons the statistics are based on and the mean number of annihilation events for the given histogram. The dashed arrows indicate the main changes upon increasing the exciton density. The upper panel does not refer to any exciton density in experiment, but was added to illustrate 20% probability of EEA even at exciton densities several times lower than experimentally used.

Supplementary Figure 13 depicts histograms for the number of annihilation events that excitons accumulated during the simulation of isolated inner tubes for a range of exciton densities. Increasing the exciton density leads to more prominent EEA. As a result, there is a lower number of excitons that decay naturally, which is reflected in a decreasing number of excitons that did not participate in any annihilation event, i.e., the number of accumulated EEA events of zero. Simultaneously, the distribution shifts to higher numbers of EEA events, as excitons are more likely to encounter another exciton and, thus, engage in another EEA event. Expectedly, the mean number of EEA participations increases from 1.01 at one exciton per ~600 molecules up to 1.91 at one exciton per ~20 molecules for increasing exciton densities evidencing the importance of multi-annihilation events accumulated by individual excitons. Even for the lowest exciton density in experiment, MC simulations show that a considerable share of the excitons has accumulated two EEA events by the time of their death, which is the primary requirement for the observation of multi-exciton processes encoded in seventh and higher-order signals.

6.6. Exciton–Exciton Annihilation Statistics: Complete Nanotubes

In order to elucidate the fate of the excitons that were originally planted on the outer tube, we extract the fraction of these excitons that (1) decay naturally, (2) decayed due to EEA on the inner tube or (3) decayed due to EEA on the outer tube at various exciton densities as shown in Supplementary Figure 14. At low exciton densities, the inter-layer exciton transfer (ET) rate is significantly faster than the EEA rates (i.e., number of EEA events per time interval), which causes the majority of the excitons from the outer tube to be transferred first and then decay naturally or, to a lesser extent, annihilate on the inner tube (Supplementary Figure 14, red curve). At the same time, EEA on the outer tube is negligibly small (Supplementary Figure 14, black curve). In contrast, for very high exciton densities, the EEA rate accelerates and ultimately outcompetes the ET rate, which leads to very prominent EEA on the outer tube. As a result, the exciton population is depleted before any of the surviving excitons can be transferred to the inner tube for which EEA is then less pronounced. At intermediate exciton densities, the ET rate and EEA rate are balanced, which maximizes the share of excitons that were planted on the outer tube, but annihilate on the inner tube. Note that Figure 6 in the main text is a sum of the excitons that either decayed naturally (Supplementary Figure 14; green squares) or annihilated (Supplementary Figure 14; red triangles) on the inner tube.

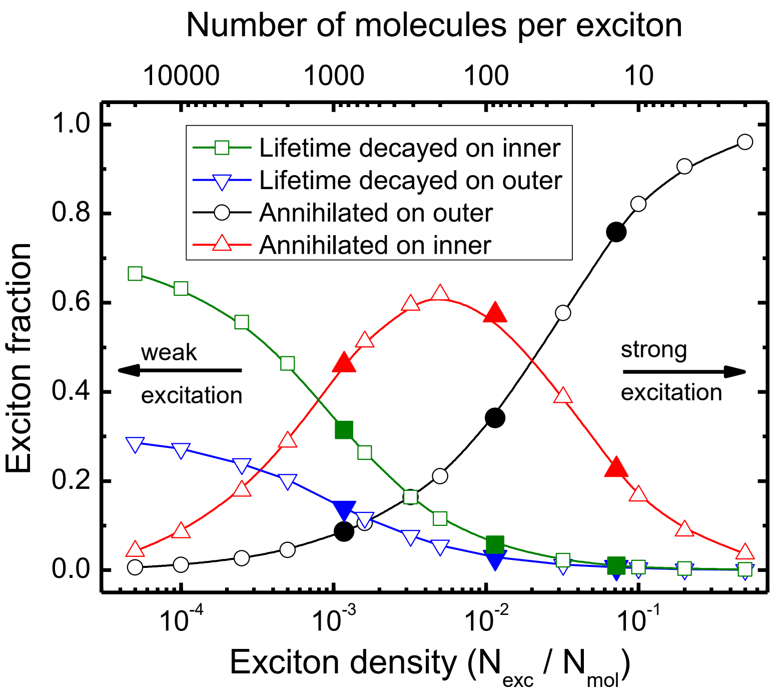

Supplementary Figure 14. Fraction of excitons that were originally planted on the outer tube that decay naturally on the inner tube (green squares), decay naturally on the outer tube (blue triangles), annihilate on the inner tube (red triangles) or annihilate on the outer tube (black circles) from the Monte-Carlo simulations as a function of the exciton density. At time zero both tubes are populated with the same exciton density. The inverse exciton density, i.e., the number of molecules per exciton is plotted on the top axis. Solid symbols: exciton densities used in experiment on complete nanotubes; open symbols: additional data points for illustration of the trend. Solid lines are drawn to guide the eye of the reader.

In the following we analyze the distribution and the mean number of EEA events accumulated by individual excitons for which the corresponding histograms are shown in Supplementary Figure 15. Here, we evaluate the share of excitons that were planted on the outer tube, and died on either the inner (upper panels; red) or the outer tube (bottom panels; black) at the exciton densities used in experiment.

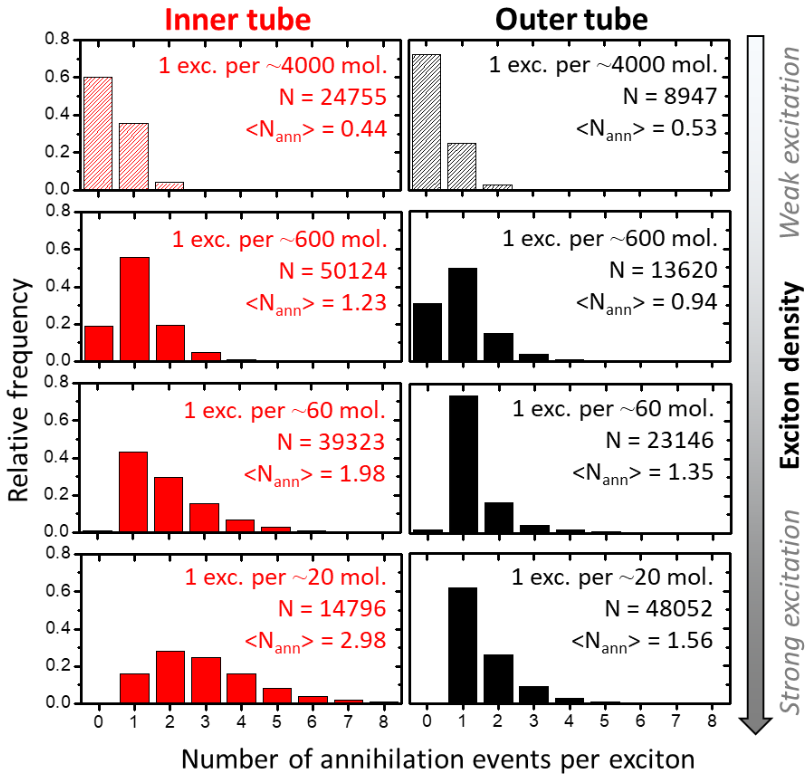

Supplementary Figure 15. Histograms of the accumulated number of annihilation events by individual excitons that were originally planted on the outer tube and died on the inner (red; left panels) or the outer tube (black; right panels) for different exciton densities. The upper panel does not refer to any exciton density in experiment, but was added to illustrate the high probability of EEA even at exciton densities ~7 times lower than experimentally used. Inset: Total number of excitons for the respective histogram and mean number of annihilation events per exciton.

For higher exciton densities EEA gains importance at expense of excitons that decay naturally before engaging in any EEA event, i.e., zero annihilation events. Consequently, the distribution of the number of EEA participations gradually shifts to higher values, where the mean number of annihilation events dramatically increases (from 1.23 to 2.98) for the inner tube, while there is only a moderate increase (from 0.53 to 1.56) for the outer tubes. Note that the upper boundary for the mean value of 2 (as discussed in the previous section) is no longer applicable here, as the

inner tube does not represent a closed system anymore, but can receive additional excitons from the outer tube. In the regime of high exciton densities, the first cascade of annihilation events occurs on the outer tube during which excitons participate in one or two EEA events ($< N_{\mathrm{ann}} >$ = 1.56). After the subsequent transfer of the surviving excitons to the inner tube, these excitons can engage in further EEA events, which leads to the large mean value of EEA participations on the inner layer, although EEA is more prominent on the outer tube in terms of total number of annihilated excitons.

Supplementary Note 7: Observation of the Seventh-Order Signal

In the Monte-Carlo simulations, we implicitly included the occurrence of multi-exciton processes, where excitons could participate in multiple exciton–exciton annihilation events. These higher-order effects (in the language of nonlinear optics, seventh-order, *etc.*; see Supplementary Note 5.3) lead to additional changes of the observed dynamics of the absorptive and EEI signals. Experimentally the seventh-order signal could also be observed despite undersampling of the coherence time with the step size of 0.38 fs (see Methods section in the main text). The latter determined the Nyquist limit at about 44000 cm^{-1}, which lies below the expected position of the seventh-order signal at triple the fundamental frequency (~50000 cm^{-1}). However, due to back folding at the Nyquist limit the seventh-order signal appeared at (44000 - 6000) cm^{-1} = 38000 cm^{-1} along the excitation axis[8]. For high exciton densities this signal was indeed resolved in the EEI signal (Supplementary Figure 16), which corroborates the influence of multi-exciton processes for the experimentally observed signals as well as in our modelling.

The seventh-order signals are observed for both complete nanotubes as well as isolated inner tubes, but significantly stronger for the former. The structure of the seventh-order signals is identical to the lower-order signals with diagonal peaks for the outer and inner tube as well as a cross peak. The only difference is that the peak signs are again inverted compared to the EEI signal and, thus, identical to the absorptive signal, where the ground-state bleach (GSB) shows up negative and excited state absorption (ESA) positive. This again originates from the two additional interactions of the sample with the incident light fields in the perturbative expansion. A quantitative analysis of these signals, however, is hindered due to the low signal amplitudes as well as the multitude of involved processes and, therefore, is beyond the scope of this paper.

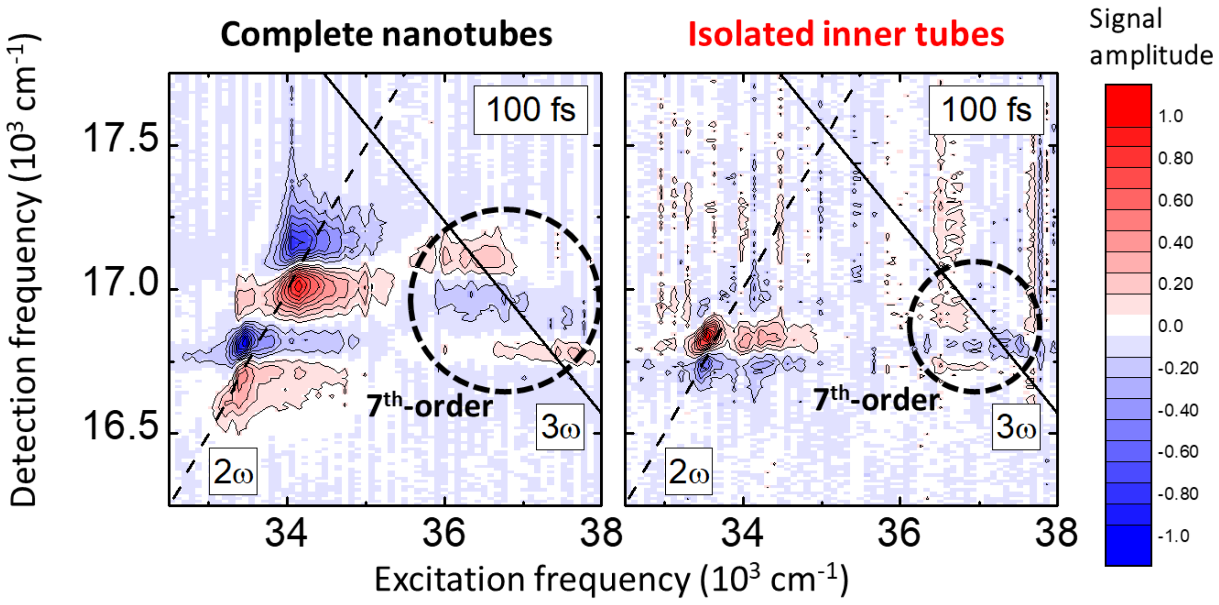

Supplementary Figure 16. EEI2D spectra of complete nanotubes (left) and isolated inner tubes (right) with the excitation axis extended to higher frequencies. The spectra were normalized to their maximum absolute amplitude. The signal amplitude is depicted on a linear color scale (between -1 and +1) with increments of 0.1 and contour lines are drawn as specified in the color bar. The 2D spectra were obtained at the highest exciton density in experiment for a waiting time of 100 fs. Dashed diagonal lines are drawn at $\omega_{excitation} = 2\omega_{detection}$. The anti-diagonal lines (solid) at 3ω originate from back folding of the diagonal $\omega_{excitation} = 3\omega_{detection}$ line at the Nyquist limit (~44000 cm^{-1}).

Supplementary Note 8: Thermal Heating Induced by Exciton–Exciton Annihilation

In the absorptive 2D spectra of complete nanotubes an interesting side effect of exciton–exciton annihilation was observed, which was reflected in transient heating of the nanotubes and a few surrounding solvent layers. The mechanism is the following: The energy of the annihilated exciton is transferred via a number of (vibrational) relaxation steps to low-frequency modes thereby creating a quasi-equilibrium Boltzmann distribution at elevated temperature. The whole relaxation process takes only a few ps which might be related to ultrafast cooling in liquid water[22,23]. The increased temperature leads to small but detectable modifications of the nanotubes' absorption spectrum, which causes a TA signal offset without detectable temporal variation on the time scale up to 100 ps especially evident at high exciton densities. The signal offset vanishes before the arrival of the next laser pulse, i.e., after 1 ms.

In order to investigate this effect in greater depth we have performed a series of transient absorption (TA) measurements with an extended scanning range of the delay time *T*. Here we indeed find a prominent signal offset for high exciton densities (a representative TA map is shown in Supplementary Figure 17a). In order to analyze the signal offset, we average the TA signal for delay times between 90 ps and 100 ps (Supplementary Figure 17a, side panel) and over 50 cm^{-1} along the detection axis. Specifically, the intervals for averaging are 16660 cm^{-1} to 16710 cm^{-1} and 16950 cm^{-1} to 17000 cm^{-1} for the inner and outer tube, respectively. As a next step, the signal amplitudes are plotted as a function of the respective exciton density in experiment (Supplementary Figure 17b, circles). For comparison, the signal at zero delay time is

extracted in the same spectral intervals and shown in the same graph (Supplementary Figure 17b, squares).

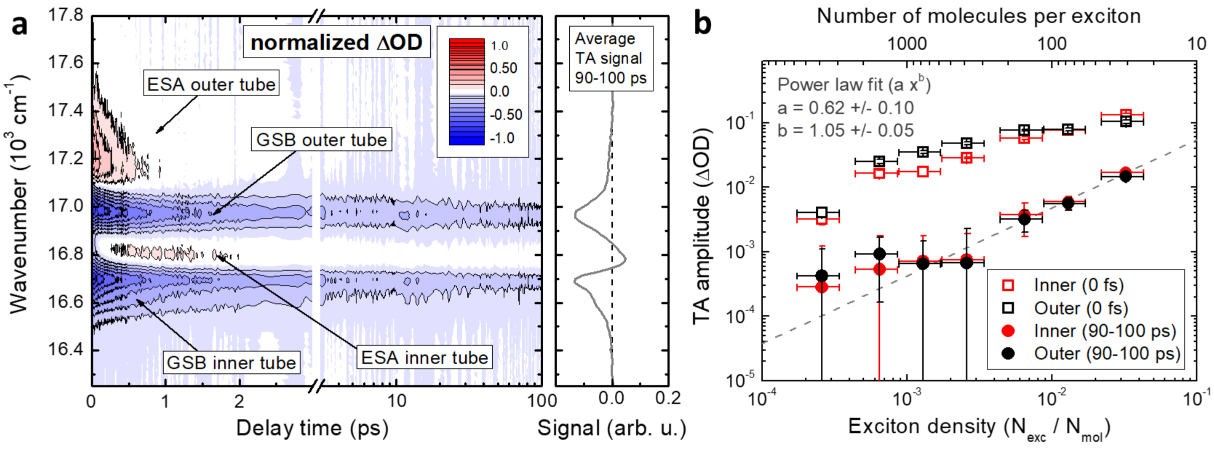

Supplementary Figure 17. (**a**) Transient absorption map of complete nanotubes for the highest exciton density (one exciton per ~30 molecules) normalized to the initial maximum absolute amplitude of the signal, i.e., change of optical density (ΔOD). The latter is depicted on a color scale (between -1 and +1) with increments of 0.1 and contour lines drawn as specified in the color bar. The vertical and horizontal axes are detection wavenumber and delay time between pump and probe pulse, respectively. The right panel depicts the average TA spectrum between 90 to 100 ps. (**b**) Log-log plot of the TA amplitude at delay times of 0 fs (open squares) and averaged between 90 ps and 100 ps (solid circles) for different exciton densities (average number of excitons per molecule) for the inner and outer tube in red and black, respectively. The top axis depicts the inverse exciton density, i.e., the number of molecules per one exciton, for simple comparison with the main text. The error bars in the vertical direction refer to the standard error upon averaging multiple scans. The horizontal error bars depict the uncertainty of the exciton density.

The long-time offset of the TA signal (Supplementary Figure 17b, solid circles) scales linearly with the exciton density, while the truly non-linear, early-time signal has a saturation scaling (Supplementary Figure 17b, open squares). These are strong indications for heating of the sample, since the amount of energy dissipated into the system scales linearly with the excitation power. This linear scaling is evident from fitting of the offset amplitude with a power

law ($f(x) = ax^b$; Supplementary Figure 17b, gray dashed line), which yields a near-unity exponent of $b = 1.05 \pm 0.05$. Furthermore, the amplitude of the offset exceeds the amplitude expected from a simple exponential decay of the TA signal, which indicates that the detected signal does not directly originate from the remaining exciton population at this delay time. According to the one-exciton lifetime of 33 ps for complete nanotubes, the signal is expected to decay to about 6 mOD (i.e., 5% of its initial amplitude ~130 mOD) after 100 ps, which is significantly lower than the measured value of ~17 mOD.

As a further test of this hypothesis we collected absorption spectra induced by a temperature jump, i.e., a change in temperature (black and orange curves in Supplementary Figure 18a). The spectra were measured by taking consecutive absorption spectra between which the sample was heated by $\Delta T = 2$ K and then allowed to slowly cool down to room temperature (RT) again. Throughout the measurement the sample temperature was monitored using a thermocouple submerged into the sample solution. The difference spectra were then computed as $\Delta\text{OD} = \text{OD}(\text{RT} + \Delta T) - \text{OD}(\text{RT})$ and depicted as black ($\Delta T = 2$ K) and orange ($\Delta T = 0.5$ K) curves in Supplementary Figure 18 in comparison with the TA "offset" spectrum at a delay time of 100 ps (blue).

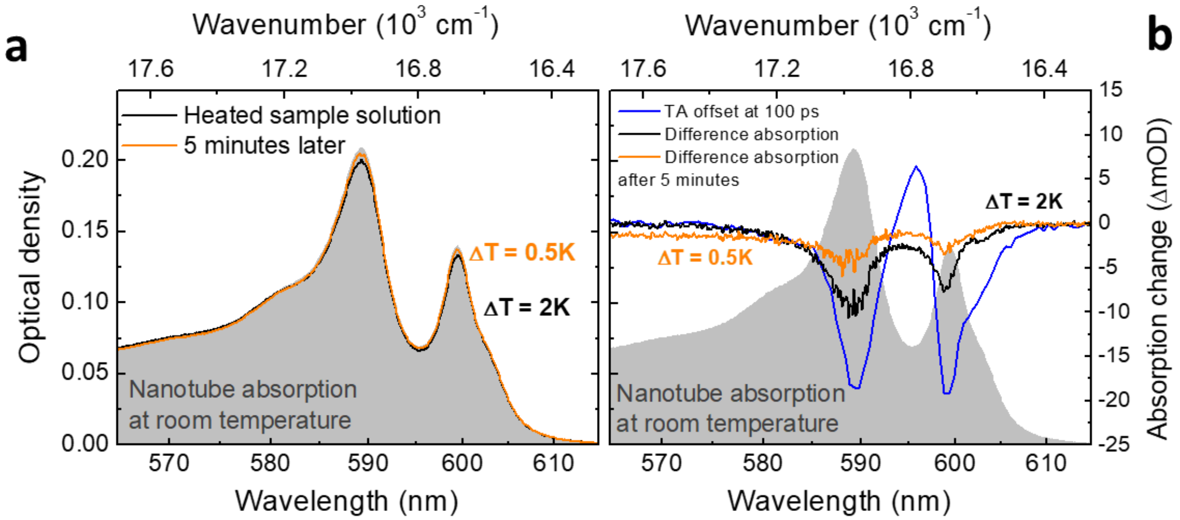

Supplementary Figure 18. (**a**) Absolute absorption spectra of complete nanotubes at room temperature (gray, shaded) and at increased temperatures ΔT = 2 K (black) and ΔT = 0.5 K (orange) after 5 min of waiting. (**b**) Pump–probe (TA) spectrum (blue) and difference spectra between C8S3 nanotube absorption spectra with a temperature difference of 2 K (black) and 0.5 K (orange).

Overall, the agreement of the TA offset and the difference spectra due to temperature regarding the spectral shape and peak positions is good with the exception of the magnitude being slightly underestimated so that a temperature change of 4 K would have likely been a better estimate. Nevertheless, these results strongly suggest that the offset observed in the absorptive 2D and TA experiments is likely to originate from transient heating of the sample.

A temperature change of 4 K corresponds to the heating due to a single laser shot (ΔE = 2.5 nJ) from which 15% are absorbed and subsequently converted into heat. To reach this estimate a number assumptions have to be made: (1) the heated volume is confined to the nanotube volume, (2) heat dissipation into the bulk solvent is negligible at the time scale of the experiment (~100 ps), and (3) the heat capacity of the nanotubes is identical to water (specific heat capacity c_s =

4.1379 J g^{-1} K^{-1} at room temperature), as the heat capacity of the nanotubes is unknown. The change in temperature is then computed via

$$\Delta T = \frac{Q}{C} = \frac{A\,\Delta E}{c_\mathrm{s}\, m_{\mathrm{H_2O}}} = \frac{0.15 \times 2.5 \times 10^{-9}\ \mathrm{J}}{4.1379 \frac{\mathrm{J}}{\mathrm{g\,K}} \times 2.2 \times 10^{-11}\ \mathrm{g}} \approx 4\ \mathrm{K} \quad (4)$$

with the supplied heat Q, absorbed fraction of the laser pulse A, pulse energy ΔE, and the heat capacity C. The corresponding mass of water was calculated from that fraction of the focal volume V_{foc} that is actually occupied by nanotubes. Therefore, the latter were treated as simple hollow cylinders with an inner and outer radius of $R_{\mathrm{inner}} = 3.551$ nm and $R_{\mathrm{outer}} = 6.465$ nm, respectively. The focal volume is assumed cylindrical with a radius of 50 µm and thickness of 50 µm yielding for the water mass

$$m_{\mathrm{H_2O}} = \left(\frac{c\, N_A\, V_{\mathrm{foc}}}{\rho_{\mathrm{total}}^{\mathrm{mol}}}\right) \pi \left(R_{\mathrm{outer}}^2 - R_{\mathrm{inner}}^2\right) \rho_{\mathrm{H_2O}} \approx 2.2 \times 10^{-11}\ \mathrm{g}. \quad (5)$$

The first bracketed factor computes the total length of nanotubes in the focal volume via the molar concentration $c = 1.11 \times 10^{-4}$ M (Methods and Supplementary Note 1), Avogadro constant N_A, and the molar density $\rho_{\mathrm{total}}^{\mathrm{mol}} = 114\ \mathrm{nm}^{-1}$ (number of molecules per unit length of the nanotubes counting both layers; extracted from the theoretical model presented in ref. 2). Taken together with the cross section of the nanotube calculated from the inner and outer radii and the density of water ($\rho_{\mathrm{H_2O}}$) yields the corresponding mass. Note that if the entire focal volume (V_{foc}) is assumed to heat up, the absolute change in temperature ΔT is in the sub-mK range.

Supplementary Note 9: Photoluminescence (PL) Measurements for One-Exciton Lifetime

In order to accurately determine the one-exciton lifetime of complete nanotubes as well as flash-diluted inner tubes we measured the photoluminescence (PL) response of the sample at extremely low exciton densities of about one exciton per 10^4 molecules for which the transients are shown in Supplementary Figure 19. In either case the sample was excited at 550 nm and the PL response was recorded with a streak camera (Hamamatsu, model C5680).

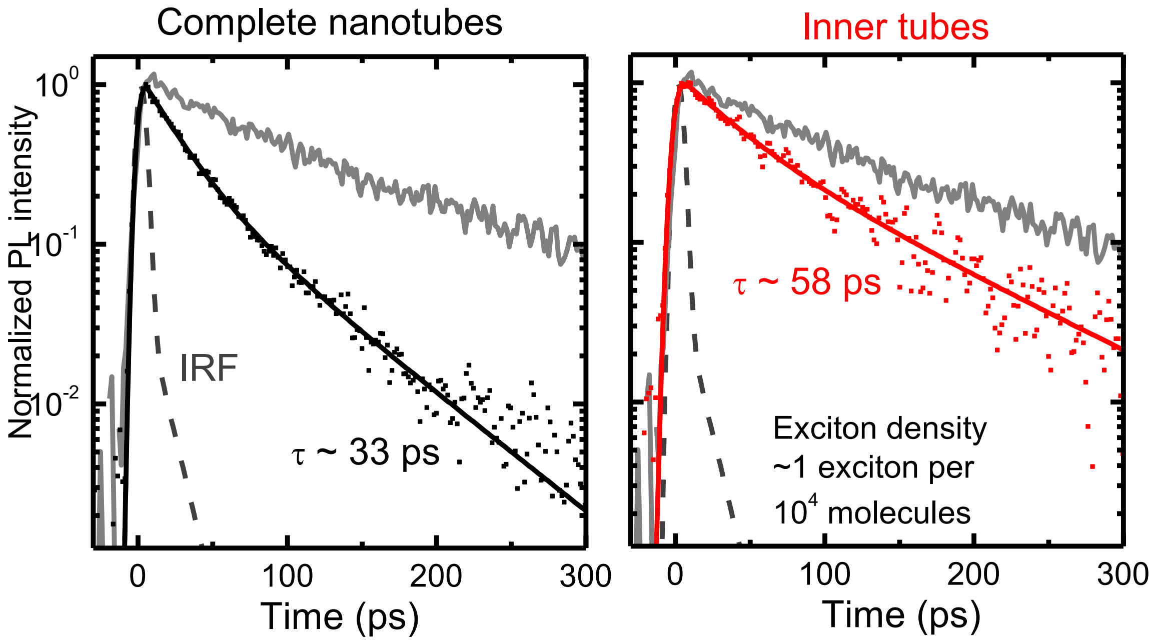

Supplementary Figure 19. Experimental PL transients for complete nanotubes (black dots) and isolated inner tubes (red dots) at low exciton densities of only one exciton per 10^4 molecules. The excitation wavelength is 550 nm. The solid lines are fits according to a bi-exponential decay convoluted with the instrument response function (IRF, dashed). Gray line: PL decay of C8S3 monomers dissolved in methanol (lifetime ~100 ps).

Despite the low exciton density, we find that the transients exhibit a small degree of non-exponentiality. Therefore, we fit the transients with a convolution of two exponential decays and the instrument response function (IRF), which can be approximated by a Gaussian with standard deviation width of ~3 ps. From these fits we extract the weighted averaged lifetime of a single exciton for the isolated inner tubes and complete nanotubes as 58 ps and 33 ps, respectively. In either case the lifetime is shorter than for C8S3 monomers dissolved in methanol (~100 ps) due to the formation of a super-radiant state[5].

The lifetime of complete nanotubes is in good agreement with previously published values obtained from femtosecond transient grating photoluminescence measurements obtained for nanotubes suspended in a sugar matrix following 400 nm excitation[7]. It is worth noting that some studies reported PL lifetimes of ~64 ps at 100 K by freezing the nanotubes in their aqueous host solvent[24] and up to ~260 ps at room temperature for nanotubes suspended in a sugar matrix[25]. The cause for these differences is not exactly understood, although the choice of the host matrix as well as the experimental parameters such as excitation wavelengths (495 nm in ref. 24; 400 nm, and 520 nm in ref. 25) might play a role.

The reduced lifetime of complete nanotubes compared to the isolated inner tubes is consistent with earlier reported observations for matrix-suspended oxidized nanotubes[7]. We hypothesize that the presence of the outer tube may introduce additional non-radiative pathways through which inner-tube excitons decay.

Supplementary Note 10: Exciton Diffusion Tensor using the Haken-Strobl-Reineker Model

10.1. Parametrization

For the molecular structure of C8S3 nanotubes, the same model (extended herringbone model with two molecules per unit cell) and parameters as reported in Eisele *et al.* are used[2]. To calculate the diffusion constant predicted by the Haken-Strobl-Reineker model of thermal fluctuations according to the expression given in the Methods section, it is necessary to know the exciton states and their corresponding energies. These are obtained for each one of the two walls separately by numerically diagonalizing the respective Hamiltonian:

$$\mathrm{H} = \sum_{n=1}^{N} \varepsilon_n \mathrm{b}_n^{\dagger} \mathrm{b}_n + \sum_{n=1}^{N} \sum_{\substack{m=1 \\ n \neq m}}^{N} J_{nm} \mathrm{b}_n^{\dagger} \mathrm{b}_m. \tag{6}$$

Here, ε_n corresponds to the excitation energy of molecule *n*, which is taken from a Gaussian distribution with mean $\varepsilon_\mathrm{o} = 18868\ \mathrm{cm}^{-1}$ and standard deviation $\sigma = 250\ \mathrm{cm}^{-1}$ in order to account for static disorder. These energetic parameters are the same as previously reported in ref. 2. Furthermore, $\mathrm{b}_n^{\dagger}(\mathrm{b}_n)$ denote the Pauli operators[26] for the creation (annihilation) of an excitation on molecule *n*. The intermolecular couplings J_{nm} are calculated using extended dipole-dipole interactions[2] and are assumed to be non-fluctuating quantities. The number of molecules is set to $N_\mathrm{inner} = 7992$ for the inner wall and $N_\mathrm{outer} = 14260$ for the outer wall, which corresponds to tubes with almost equal length of approximately $L \approx 197$ nm in accordance with ref. 2. From these values a molecular surface density of 1.81 molecules nm^{-2} was extracted as $\rho = N/2\pi RL$, where N is the total number of molecules in each tube and R is

the radius of the tube. Furthermore, the inter-tube interactions are neglected in the simulation. Under this premise the only two remaining parameters to calculate the diffusion constant tensor are the dephasing rate Γ that characterizes the thermal white noise fluctuations of the Haken-Strobl-Reineker model and the temperature T. Here, we use $\Gamma = 83.75\ \mathrm{cm}^{-1}$, which represents the average of the Lorentzian lineshape widths (half width half maximum) used in ref. 2 and T = 295 K, i.e., room temperature. We note that as seen in Supplementary Figure 20 the diffusion constants are only weakly dependent on Γ in the relevant parameter regime.

10.2. Diffusion Tensor Elements

As a result of the calculation, we obtain the diffusion tensor elements shown in Supplementary Figure 20 as a function of the dephasing rate Γ.

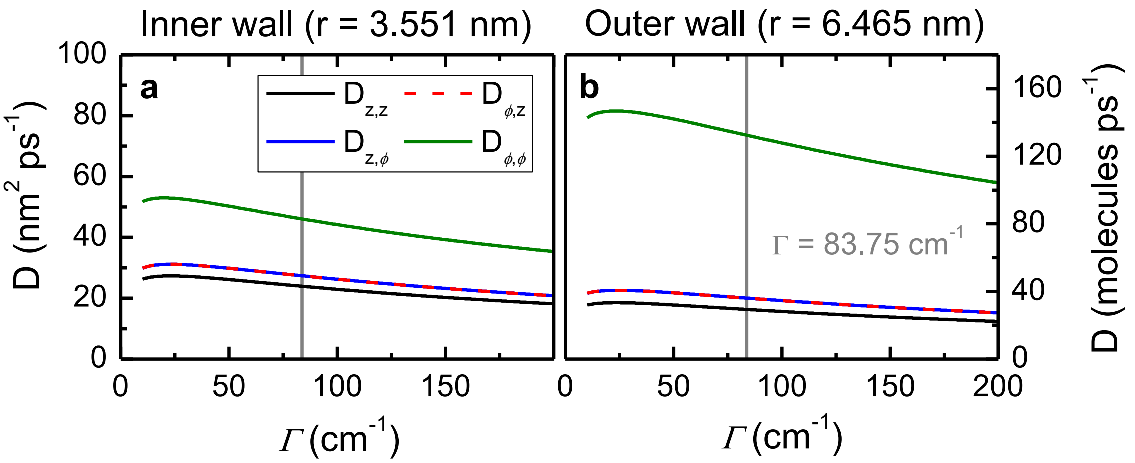

Supplementary Figure 20. Diffusion tensor elements as a function of the dephasing rate Γ for (**a**) the inner and (**b**) outer wall of double-walled nanotubes in units of $nm^2\ ps^{-1}$ (left axis) and molecules ps^{-1} (right axis). The temperature in calculations was set to 295 K and the static Gaussian energy disorder strength $\sigma = 250\ \mathrm{cm}^{-1}$. The vertical line (gray) marks the dephasing rate Γ relevant to the nanotubes.

We use the axial component ($D_{z,z}$) of the diffusion constant tensor for comparison to the results from our experiments and Monte-Carlo simulations. We justify this choice by the high aspect ratio of the nanotubes (i.e., ~20 in the MC simulations) for which $D_{z,z}$ is expected to be the dominant component for exciton–exciton annihilation in particular for longer diffusion times, whereas exciton diffusion around the tube ($D_{\phi,\phi}$) is less important. This assumption holds for any exciton density, as excitons on the perimeter would rapidly annihilate after which the later dynamics are again governed by the axial diffusion constant. The different elements of the diffusion constant tensor at a given dephasing rate of $\Gamma = 83.75\ \mathrm{cm}^{-1}$ (HWHM) are summarized in Supplementary Table 4.

Supplementary Table 4. Individual elements of the diffusion constant tensor for a dephasing rate of $\Gamma = 83.75\ \mathrm{cm}^{-1}$ (HWHM) for the inner and outer tube.

	Inner tube	Outer tube
$D_{z,z}$	$23.9\ \mathrm{nm}^2\ \mathrm{ps}^{-1}$	$16.3\ \mathrm{nm}^2\ \mathrm{ps}^{-1}$
$D_{\phi,z} = D_{z,\phi}$	$27.4\ \mathrm{nm}^2\ \mathrm{ps}^{-1}$	$20.0\ \mathrm{nm}^2\ \mathrm{ps}^{-1}$
$D_{\phi,\phi}$	$46.0\ \mathrm{nm}^2\ \mathrm{ps}^{-1}$	$73.6\ \mathrm{nm}^2\ \mathrm{ps}^{-1}$

Supplementary Note 11: Monte-Carlo Simulations for Purely Diffusive Exciton Dynamics

Here we test a scenario in which the exciton–exciton interaction radius is nullified, i.e., two excitons only annihilate in case they occupy the same site after a hopping event, which is compensated by an increased hopping rate to obtain a diffusion constant of 100 nm^2 ps^{-1} in accordance with previously published results[27]. All other conditions as outlined in the main text, stayed unaltered.

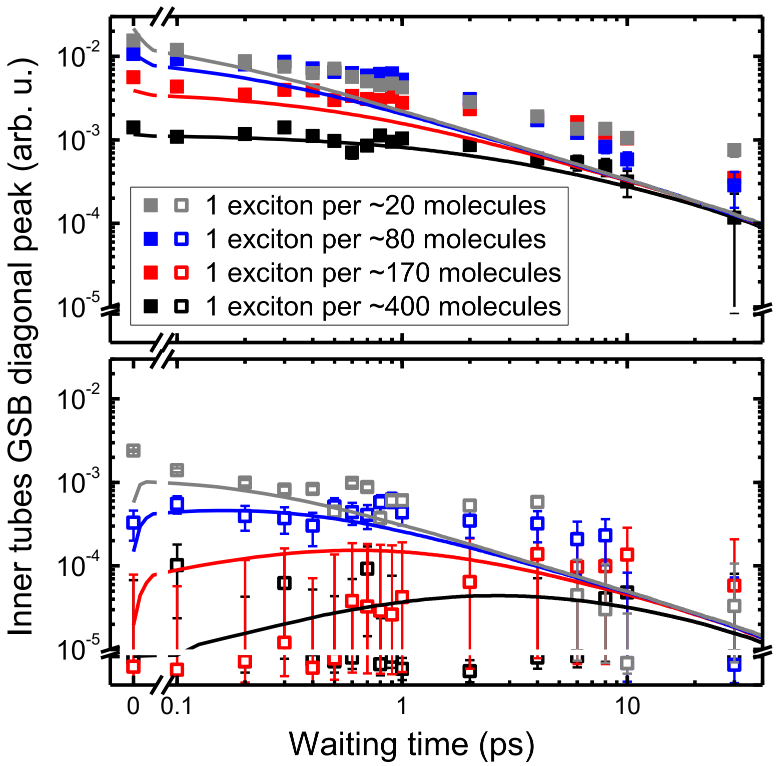

Supplementary Figure 21. Experimental absorptive and EEI transients for isolated inner tubes as a function of waiting time (symbols). The experimental data are the same as in Figure 3 in the main paper. The solid lines depict transients from MC simulations.

In the regime of high exciton densities (Supplementary Figure 21, gray), the dynamics at early waiting times are captured reasonably well, as a faster exciton diffusion can compensate the lack of an extended annihilation radius and *vice versa*. However, towards longer waiting times the increased diffusion constant leads to unsatisfactory fit of the data, as the calculated dynamics are generally too fast. In particular, this trend becomes apparent at intermediate exciton densities, where the simulations predicts the maximum EEI signal to occur at ~600 fs (Supplementary Figure 21, red) and ~200 fs (Supplementary Figure 21, blue), although the experimental data reach the maximum amplitude at ~6 ps and ~1 ps, respectively. Therefore, we conclude that exciton diffusion alone cannot account for the experimental observations, but an extended radius for exciton–exciton interactions is required to describe the data adequately.

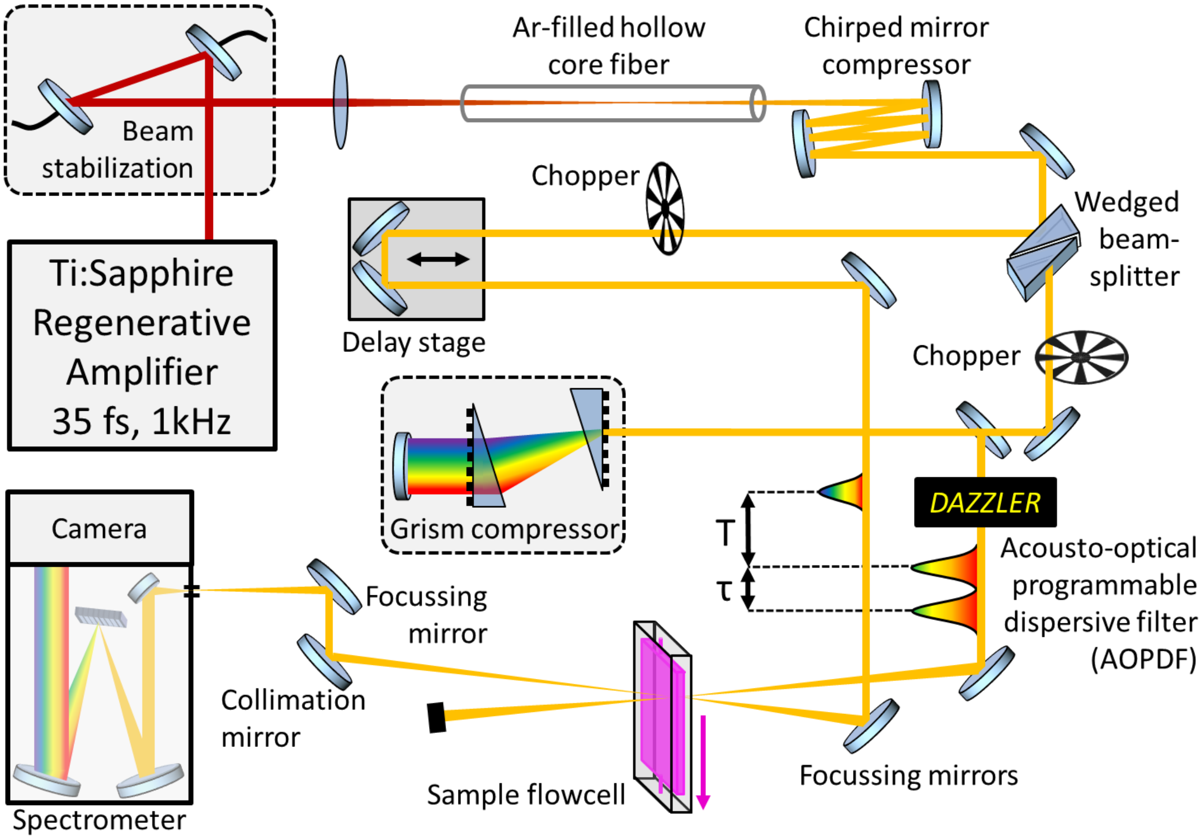

Supplementary Figure 22. Schematic of the experimental apparatus for absorptive 2D and EEI2D spectroscopy. The flow direction of the sample is indicated by the arrow. The pump and probe pulses are polarized along the flow.

Supplementary Note 12: EEI2D Spectra on Laser Dye Sulforhodamine 101

As a control experiment, absorptive 2D and EEI2D spectra of the laser dye sulforhodamine 101 (SR101, Radiant Dyes) diluted in water at concentration of 10^{-4} mol L^{-1} were recorded (Supplementary Figure 23) at which the average distance between individual molecules amounts to ~26 nm. SR101 was chosen as its absorption peak is located in the same spectral range as the nanotube absorption spectrum investigated here. The response from diluted SR101 molecules is expected to be annihilation-free, as the individual molecules are spaced far apart and, thus, energetically uncoupled. Therefore, any photo-excitation remains localized on a single molecule, which prevents exciton–exciton annihilation. The optical density was set at OD ≈ 0.08 at 586 nm, which is similar to the OD of the nanotubes sample; the excitation energy was set at 40 nJ per pulse. This resulted in excitation of approximately 10% of the SR101 molecules in the focal volume, or, in terms of the main text, one excitation per 10 molecules. This exceeds the highest exciton density used for 2D spectroscopy of the nanotubes by a factor of ~2.

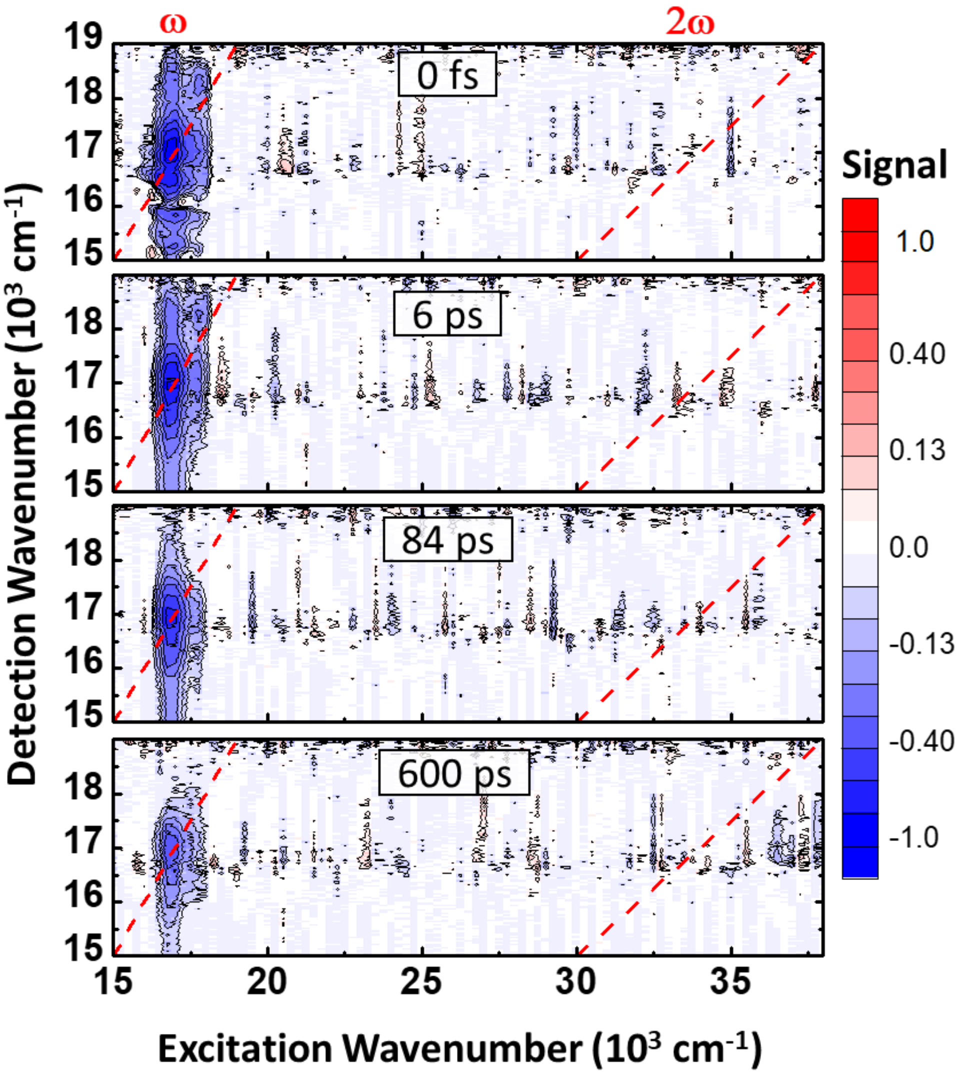

Supplementary Figure 23. Absorptive 2D and EEI2D spectra of sulforhodamine 101 dissolved in H_2O for different waiting times for an excitation power of 40 nJ. The signal amplitude was normalized to the maximum absolute amplitude (at 0 fs waiting time) and is depicted on a color scale (between -1 and +1) with increments at 0.83, 0.57, 0.4, 0.27. 0.19, 0.13, 0.08, 0.05. Contour lines are drawn as specified in the color bar. Dashed lines (red) are drawn at $\omega_{excitation} = \omega_{detection}$ and $\omega_{excitation} = 2\omega_{detection}$ to mark the positions of the absorptive and EEI signals, respectively.

The 2D absorptive response is governed by a broad (negative) ground-state bleach GSB and stimulated emission (SE) signal around the fundamental frequency ω, while there is no EEI signal detectable around the double frequency 2ω. Furthermore, Supplementary Figure 23

depicts the full range of excitation frequencies from 15000 cm^{-1} to 36000 cm^{-1} in order to prove that the signal at intermediate frequencies is free of any artifacts or spurious signals. These results confirm that the spectral range, where EEI signals are expected, is free from artifacts from the experimental apparatus. Importantly, it also supports our assignment of the EEI signal arising from exciton–exciton annihilation, because an even higher excitation density for dissolved sulforhodamine 101 molecules does not result in any observable EEI signal, which in turn justifies our theoretical approach.

Supplementary Note 13: Inter-Wall Excitation Transfer Rate

In order to determine the transfer rate from the outer to the inner tube for complete nanotubes, absorptive 2D spectroscopy was used with fine sampling of the waiting time in steps of 5 fs. To minimize the influence of exciton–exciton annihilation, the exciton density was set to only one exciton per ~500 molecules, which caused the increased noise level of the transient. Supplementary Figure 24 depicts the ground-state bleach (GSB) cross peak amplitude as a function of waiting time.

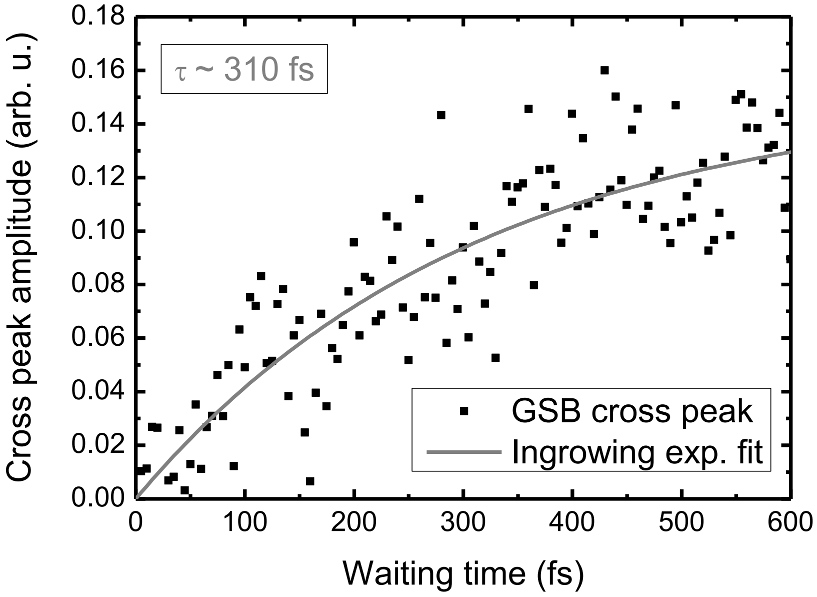

Supplementary Figure 24. GSB cross peak amplitude of complete nanotubes as a function of waiting time (black squares). The gray line is an exponential fit of the transient.

Fitting the GSB cross peak transient with an in-growing exponential function $F(t) = A[1 - \exp(-t/\tau)]$ yields a time constant of ~310 fs, which is in excellent agreement with the values reported in literature[7,28,29].

Supplementary References

1. Dostál, J.; Fennel, F.; Koch, F.; Herbst, S.; Würthner, F.; Brixner, T. Direct observation of exciton–exciton interactions. *Nat. Commun.* **9,** 2466 (2018).

2. Eisele, D. M.; Cone, C. W.; Bloemsma, E. A.; Vlaming, S. M.; van der Kwaak, C. G. F.; Silbey, R. J.; Bawendi, M. G.; Knoester, J.; Rabe, J. P.; Vanden Bout, D. A. Utilizing redox-chemistry to elucidate the nature of exciton transitions in supramolecular dye nanotubes. *Nat. Chem.* **4,** 655–662 (2012).

3. Kriete, B.; Bondarenko, A. S.; Jumde, V. R.; Franken, L. E.; Minnaard, A. J.; Jansen, T. L. C.; Knoester, J.; Pshenichnikov, M. S. Steering Self-Assembly of Amphiphilic Molecular Nanostructures via Halogen Exchange. *J. Phys. Chem. Lett.* **8,** 2895–2901 (2017).

4. Clark, K. A.; Cone, C. W.; Vanden Bout, D. A. Quantifying the Polarization of Exciton Transitions in Double-Walled Nanotubular J-Aggregates. *J. Phys. Chem. C* **117,** 26473–26481 (2013).

5. Spano, F. C.; Mukamel, S. Superradiance in molecular aggregates. *J. Chem. Phys.* **91,** 683–700 (1989).

6. Igor, S.; Tobias, B.; Mino, Y.; Fleming, G. R. Heterogeneous Exciton Dynamics Revealed by Two-Dimensional Optical Spectroscopy. *J. Phys. Chem. B* **110,** 20032–20037 (2006).

7. Pandya, R.; Chen, R.; Cheminal, A.; Thomas, T. H.; Thampi, A.; Tanoh, A.; Richter, J. M.; Shivanna, R.; Deschler, F.; Schnedermann, C.; *et al.* Observation of Vibronic Coupling Mediated Energy Transfer in Light-Harvesting Nanotubes Stabilized in a Solid-State

Matrix. *J. Phys. Chem. Lett.* **9,** 5604–5611 (2018).

8. Hamm, P.; Zanni, M. *Concepts and Methods of 2D Infrared Spectroscopy*. (Cambridge University Press, 2011).

9. Mukamel, S. *Principles of Nonlinear Optical Spectroscopy*. (Oxford University Press, Oxford, 1995).

10. Süß, J.; Wehner, J.; Dostál, J.; Brixner, T.; Engel, V. Mapping of exciton–exciton annihilation in a molecular dimer via fifth-order femtosecond two-dimensional spectroscopy. *J. Chem. Phys.* **150,** 104304 (2019).

11. Malý, P.; Mančal, T. Signatures of Exciton Delocalization and Exciton–Exciton Annihilation in Fluorescence-Detected Two-Dimensional Coherent Spectroscopy. *J. Phys. Chem. Lett.* **9,** 5654–5659 (2018).

12. von Berlepsch, H.; Kirstein, S.; Hania, R.; Pugzlys, A.; Böttcher, C.; Pugžlys, A.; Böttcher, C.; Pugzlys, A.; Böttcher, C. Modification of the nanoscale structure of the J-aggregate of a sulfonate-substituted amphiphilic carbocyanine dye through incorporation of surface-active additives. *J. Phys. Chem. B* **111,** 1701–1711 (2007).

13. Qiao, Y.; Polzer, F.; Kirmse, H.; Kirstein, S.; Rabe, J. P. Nanohybrids from nanotubular J-aggregates and transparent silica nanoshells. *Chem. Commun.* **51,** 11980–11982 (2015).

14. Didraga, C.; Pugžlys, A.; Hania, P. R.; von Berlepsch, H.; Duppen, K.; Knoester, J. Structure, spectroscopy, and microscopic model of tubular carbocyanine dye aggregates. *J. Phys. Chem. B* **108,** 14976–14985 (2004).

15. Sperling, J.; Nemeth, A.; Hauer, J.; Abramavicius, D.; Mukamel, S.; Kauffmann, H. F.; Milota, F. Excitons and disorder in molecular nanotubes: a 2D electronic spectroscopy study and first comparison to a microscopic model. *J. Phys. Chem. A* **114,** 8179–8189 (2010).

16. Megow, J.; Röhr, M. I. S. S.; Schmidt am Busch, M.; Renger, T.; Mitrić, R.; Kirstein, S.; Rabe, J. P.; May, V.; am Busch, M.; Renger, T.; *et al.* Site-dependence of van der Waals interaction explains exciton spectra of double-walled tubular J-aggregates. *Phys. Chem. Chem. Phys.* **17,** 6741–7 (2015).

17. Czikkely, V.; Försterling, H. D.; Kuhn, H. Light absorption and structure of aggregates of dye molecules. *Chem. Phys. Lett.* **6,** 11–14 (1970).

18. Förster, T. Zwischenmolekulare energiewanderung und fluoreszenz. *Ann. Phys.* **437,** 55–75 (1948).

19. Engel, E.; Leo, K.; Hoffmann, M. Ultrafast relaxation and exciton–exciton annihilation in PTCDA thin films at high excitation densities. *Chem. Phys.* **325,** 170–177 (2006).

20. Fennel, F.; Lochbrunner, S. Exciton-exciton annihilation in a disordered molecular system by direct and multistep Förster transfer. *Phys. Rev. B* **92,** 140301 (2015).

21. Mikhnenko, O. V.; Blom, P. W. M.; Nguyen, T.-Q. Exciton diffusion in organic semiconductors. *Energy Environ. Sci.* **8,** 1867–1888 (2015).

22. Yeremenko, S.; Pshenichnikov, M. S.; Wiersma, D. A. Interference effects in IR photon echo spectroscopy of liquid water. *Phys. Rev. A* **73,** 021804 (2006).

23. Lindner, J.; Vöhringer, P.; Pshenichnikov, M. S.; Cringus, D.; Wiersma, D. A.; Mostovoy, M. Vibrational relaxation of pure liquid water. *Chem. Phys. Lett.* **421,** 329–333 (2006).

24. Pugžlys, A.; Augulis, R.; Van Loosdrecht, P. H. M.; Didraga, C.; Malyshev, V. A.; Knoester, J. Temperature-dependent relaxation of excitons in tubular molecular aggregates: fluorescence decay and Stokes shift. *J. Phys. Chem. B* **110,** 20268–20276 (2006).

25. Caram, J. R.; Doria, S.; Eisele, D. M.; Freyria, F. S.; Sinclair, T. S.; Rebentrost, P.; Lloyd, S.; Bawendi, M. G. Room-Temperature Micron-Scale Exciton Migration in a Stabilized Emissive Molecular Aggregate. *Nano Lett.* **16,** 6808–6815 (2016).

26. Davydov, A. S. *Theory of Molecular Excitons*. (Plenum, 1971).

27. Clark, K. A.; Krueger, E. L.; Vanden Bout, D. A. Direct measurement of energy migration in supramolecular carbocyanine dye nanotubes. *J. Phys. Chem. Lett.* **5,** 2274–2282 (2014).

28. Augulis, R.; Pugzlys, A.; van Loosdrecht, P. H. M.; Pugžlys, A.; van Loosdrecht, P. H. M.; Pugzlys, A.; van Loosdrecht, P. H. M. Exciton dynamics in molecular aggregates. *Phys. status solidi* **3,** 3400 (2006).

29. Yuen-Zhou, J.; Arias, D. H.; Eisele, D. M.; Steiner, C. P.; Krich, J. J.; Bawendi, M. G.; Nelson, K. A.; Aspuru-Guzik, A. Coherent exciton dynamics in supramolecular light-harvesting nanotubes revealed by ultrafast quantum process tomography. *ACS Nano* **8,** 5527–5534 (2014).
