# Supplementary material for: Interplay between structural hierarchy and exciton diffusion in artificial light harvesting
Source: Nat Commun. 2019 Oct 10;10:4615. doi: 10.1038/s41467-019-12345-9 (PMC6787233; doi:10.1038/s41467-019-12345-9)
Supplement: Supplementary file 3 — Description of Additional Supplementary Files [file 41467_2019_12345_MOESM3_ESM.pdf]

Description of Additional Supplementary Files

File Name: Supplementary Software 1

Description: The supplementary software package available on the publishers website contains the compiled program for Monte-Carlo simulations (including a short description of how to operate the program) and a set of sample data.
