# Supplementary material for: Interplay between structural hierarchy and exciton diffusion in artificial light harvesting
Source: Nat Commun. 2019 Oct 10;10:4615. doi: 10.1038/s41467-019-12345-9 (PMC6787233; doi:10.1038/s41467-019-12345-9)
Supplement: Supplementary file 4 — Supplementary Software 1 [file 41467_2019_12345_MOESM4_ESM.zip › Readme.pdf]

Monte-Carlo simulations

Monte Carlo simulations were performed using home written software (XYYCalc.exe) in combination with a custom written macro (see below). The program XYYCalc runs under the operating system Microsoft Windows 7 or higher. It requires the Microsoft .NET 4.5 (or higher) FrameWork, which is already installed by default on Windows 8 or higher. On Windows 7 it can be installed as an update.

For XYYCalc no installation is needed. It can simply be unpacked in any directory. Monte-Carlo simulations of exciton dynamics on nanotubes are included in the FFBjorn.dll under “DoubleWallAggregateDiffusion”. Monte-Carlo simulations can be run by starting the program and going to “edit macro” → “load” → MonteCarloCode.txt (from the same directory as the XYYCalc program) → Execute. This would calculate the exciton dynamics for complete nanotubes for the preset parameters. The parameters can be changed in the “Par.txt” file, which needs to be placed in the directory described in the code below (default: “C:\tmp”). The exciton densities and the respective number of iterations need to be specified in separate files (“ED_complete.txt” and “ED_inner.txt”) in a separate folder “Exciton density” in the same directory. For the simulation of isolated inner tubes the lines marked in blue need to be changed with the appropriate parameters (as provided in the manuscript). Lines marked in red are not relevant to the current study.

```
//Declaration of variables
integer i, j, k, NrChannels, NrChannels2, Index1, Index2;
Integer Success;

string directory, ChannelName, p;
string directoryI, directoryC;
string directoryII, directoryCC;

float Maxs, Normfactor, A;
float HR, AR, TD, AT, LTc, LTi;
Float redChiSqr;

floatmatrix EDc, EDi, Par;

floatarray FitParameters[24];
floatArray OptimizeParameters[24];
FloatArray Lowboundaries[24];
FloatArray Highboundaries[24];
FloatArray YFitted;

//Define directory in which simulation data is supposed to be stored
directory := 'C:\tmp';
AppendBackslash(directory);

CreateDir(directory + 'complete tubes');
directoryC := directory + 'complete tubes';
appendbackslash(directoryC);

CreateDir(directory + 'inner tubes');
directoryI := directory + 'inner tubes';
appendbackslash(directoryI);
```

```
//Load parameter file that contains a matrix with simulation parameters in the respective column:
//[HoppingRate AnnihilationRadius TrapDensity AnnTrapProbablity]
Par := LoadMatrix(directory + 'Par.txt');

//Load files that contain the input exciton densities (ED)
//Each file contains three columns with [Power ED Iterations]
//EDc = exciton density complete nanotubes
//EDi = exciton density isolated inner tubes
EDc := LoadMatrix(directory + 'Exciton Density\ED_complete.txt');
EDi := LoadMatrix(directory + 'Exciton Density\ED_inner.txt');

//Run simulations for all input parameters and exciton densities
for k := 0 to Arraysize(Par[0, ])-1 do
        begin
        HR := Par[0, k]; //hopping rate
        AR := Par[1, k]; //annihilation radius
        TD := Par[2, k]; //Intensity dependent trap density
        AT := Par[3, k]; //Annihilation to trap probability
        LTc := 33000; //one-exciton lifetime complete tubes (in fs)
        LTi := 58000; //one-exciton lifetime inner tubes (in fs)

XYYdata_clear;

//Loads file to define the x-axis
XYYData_Load(directory + 'xaxis_0-500ps_25fs_steps.txt');
XYYData_SetNrChannels(2*Arraysize(EDc[0, ]));

//Define sub-directory for data file
CreateDir(directoryC + 'H=' + NumberToStr(HR) + ', Di=Do=' + NumberToStr(AR)+ ', TD=' +
NumberToStr(TD) + ', AT=' + NumberToStr(AT));
directoryCC := directoryC + 'H=' + NumberToStr(HR) + ', Di=Do=' + NumberToStr(AR)+ ', TD='
+ NumberToStr(TD) + ', AT=' + NumberToStr(AT);
appendbackslash(directoryCC);

//Run simulations for all exciton densities for complete tubes
for i:=1 to Arraysize(EDc[0, ]) do
begin

                FitParameters[0] := 1;          // Amplitude scaling factor
                FitParameters[1] := 0;          // Constant baseline
                FitParameters[2] := 0;          // Gauss X0 (for convolution of transient with
                                                 instrument response function)
                FitParameters[3] := 0;          // Gauss sigma (for convolution of transient with
                                                 instrument response function)
                FitParameters[4] := LTc;        // one-exciton lifetime (in fs)
                FitParameters[5] := 1;          // Time of 1 step in simulation (in fs)
                FitParameters[6] := HR;         // Hopping probability
                FitParameters[7] := 0.0013;     // Transfer probability (Inner to outer tube)
                FitParameters[8] := 0.0031;     // Transfer probability (Outer to inner tube)
                FitParameters[9] := 30;         // Grid: inner width(X) sites
                FitParameters[10] := 1000;      // Grid: inner length(Y) sites
                FitParameters[11] := 55;        // Grid: outer width(X) sites
                FitParameters[12] := 1000;      // Grid: outer length(Y) sites
                FitParameters[13] := EDc[1, i-1];  // Initial exciton density (inner tube)
                FitParameters[14] := EDc[1, i-1];  // Initial exciton density (outer tube)
```

```
                FitParameters[15] := AR;        // Annihilation radius (inner tube; set -1 for no
                                                  annihilation)
                FitParameters[16] := AR;        // Annihilation radius (outer tube; set -1 for no
                                                  annihilation)
                FitParameters[17] := 0;         // Excitation center time (for finite pulse length)
                FitParameters[18] := 0;         // Excitation FWHM (for finite pulse length)
                FitParameters[19] := 1;         // Annihilation probability
                FitParameters[20] := 0;         // Inner tube trap density
                FitParameters[21] := 0;         // Outer tube trap density
                FitParameters[22] := 0;         // Tube hop XY diffuse
                FitParameters[23] := AT;        // Trap formation due to annihilation

                // Monte-Carlo simulations
                Fit1D_SetFunction('Bjorn.DoubleWallAggregateDiffusion');
                Fit_SetConstants ( Round(EDc[2, i-1]), 6, 0, 0);
                //The input parameters define the number of iterations, number of CPU Cores,
                 output channel (excitons on inner layer @ time T), save statistics: 0 no, 1 yes)
                //Note that the output channel here is not relevant, as all data will be saved at the
                 end of the run.
                OptimizeParameters:= false;

                // Plotting of simulated data
                Success := Fit1D_MakeFit(XAxis, ch[1], FitParameters, YFitted, redChiSqr);

end;

XYYData_Load(directory + '__DoubleWallAggregateDiffusion_AllData.txt');
XYYData_Save(directoryCC + 'All_Data.txt');

end;
```

A typical calculation (for complete nanotubes with two sets of input parameters and a reasonable number of iterations for good statistics; between 10 and 1000 given the exciton densities in the current study) takes approximately 5 minutes using a PC with an Intel Core i7 vPro 6 cores 3 GHz 8th Gen processor.

Expected output: The output file (one for each set of input parameters and each exciton density) contains a total of 12 columns with the time axis in the first row and each of the following rows evaluating the respective quantity at time T of the simulation. The rows marked in green were used for the manuscript.

#	Quantity	Explanation
1	Time	Time axis defined by the x-axis preloaded in the macro, i.e., from 0 to 500ps in 25fs steps
2	#excitons inner tube	Total number of excitons residing on the inner tube at time T of the simulation
3	#excitons outer tube	Total number of excitons residing on the inner tube at time T of the simulation
4	#annihilation	Total number of exciton-exciton annihilation events per timestep Δt for both tubes
5	#inner->inner	Total number of excitons that were initially planted on the inner tube and reside on the inner tube at time T

6	#outer->outer	Total number of excitons that were initially planted on the outer tube and reside on the outer tube at time T
7	#inner->outer	Total number of excitons that were initially planted on the inner tube and reside on the outer tube at time T
8	#outer->inner	Total number of excitons that were initially planted on the outer tube and reside on the inner tube at time T
9	#Ann.survivor inner->inner	Total number of excitons that were initially planted on the inner tube, have participated in at least one exciton-exciton annihilation event until time T and reside on the inner tube at time T
10	#Ann.survivor outer->outer	Total number of excitons that were initially planted on the outer tube, have participated in at least one exciton-exciton annihilation event until time T and reside on the outer tube at time T
11	#Ann.survivor inner->outer	Total number of excitons that were initially planted on the inner tube, have participated in at least one exciton-exciton annihilation event until time T and reside on the outer tube at time T
12	#Ann.survivor outer->inner	Total number of excitons that were initially planted on the outer tube, have participated in at least one exciton-exciton annihilation event until time T and reside on the inner tube at time T
